# Gallium-68-labeled fibroblast activation protein inhibitor-46 PET in patients with resectable or borderline resectable pancreatic ductal adenocarcinoma: A phase 2, multicenter, single arm, open label non-randomized study protocol

**DOI:** 10.1371/journal.pone.0294564

**Published:** 2023-11-27

**Authors:** Aashna Karbhari, Sherly Mosessian, Kamaxi H. Trivedi, Frank Valla, Mark Jacobson, Mark J. Truty, Nandakumar G. Patnam, Diane M. Simeone, Elcin Zan, Tracy Brennan, Hongli Chen, Phillip H. Kuo, Ken Herrmann, Ajit H. Goenka

**Affiliations:** 1 Department of Radiology, Mayo Clinic, Rochester, Minnesota, United States of America; 2 Clinical Development, Sofie Biosciences, Dulles, Virginia, United States of America; 3 Radiopharmaceutical and Contract Manufacturing, Sofie Biosciences, Dulles, Virginia, United States of America; 4 Department of Surgery, Mayo Clinic, Rochester, Minnesota, United States of America; 5 Departments of Surgery and Pathology, NYU Langone Health, New York, New York, United States of America; 6 Department of Radiology, Weill Cornell Medicine, New York, New York, United States of America; 7 Discovery Life Sciences, Newtown, Pennsylvania, United States of America; 8 Departments of Medical Imaging, Medicine and Biomedical Engineering, University of Arizona, Tucson, Arizona, United States of America; 9 Department of Nuclear Medicine, University Hospital Essen, University of Duisburg-Essen, Essen, Germany; Affiliated Hospital of Nanjing University of Chinese Medicine: Jiangsu Province Academy of Traditional Chinese Medicine, CHINA

## Abstract

**Background:**

Pancreatic ductal adenocarcinoma (PDAC) is a lethal disease prone to widespread metastatic dissemination and characterized by a desmoplastic stroma that contributes to poor outcomes. Fibroblast activation protein (FAP)-expressing Cancer-Associated Fibroblasts (CAFs) are crucial components of the tumor stroma, influencing carcinogenesis, fibrosis, tumor growth, metastases, and treatment resistance. Non-invasive tools to profile CAF identity and function are essential for overcoming CAF-mediated therapy resistance, developing innovative targeted therapies, and improved patient outcomes. We present the design of a multicenter phase 2 study (clinicaltrials.gov identifier NCT05262855) of [^68^Ga]FAPI-46 PET to image FAP-expressing CAFs in resectable or borderline resectable PDAC.

**Methods:**

We will enroll up to 60 adult treatment-naïve patients with confirmed PDAC. These patients will be eligible for curative surgical resection, either without prior treatment (Cohort 1) or after neoadjuvant therapy (NAT) (Cohort 2). A baseline PET scan will be conducted from the vertex to mid-thighs approximately 15 minutes after administering 5 mCi (±2) of [^68^Ga]FAPI-46 intravenously. Cohort 2 patients will undergo an additional PET after completing NAT but before surgery. Histopathology and FAP immunohistochemistry (IHC) of initial diagnostic biopsy and resected tumor samples will serve as the truth standards. Primary objective is to assess the sensitivity, specificity, and accuracy of [^68^Ga]FAPI-46 PET for detecting FAP-expressing CAFs. Secondary objectives will assess predictive values and safety profile validation. Exploratory objectives are comparison of diagnostic performance of [^68^Ga]FAPI-46 PET to standard-of-care imaging, and comparison of pre- versus post-NAT [^68^Ga]FAPI-46 PET in Cohort 2.

**Conclusion:**

To facilitate the clinical translation of [^68^Ga]FAPI-46 in PDAC, the current study seeks to implement a coherent strategy to mitigate risks and increase the probability of meeting FDA requirements and stakeholder expectations. The findings from this study could potentially serve as a foundation for a New Drug Application to the FDA.

**Trial registration:**

^@^ClinicalTrials.gov identifier NCT05262855.

## Background

Pancreatic ductal adenocarcinoma (PDAC) is a treatment refractory cancer with a very high morbidity and mortality. Currently, curative-intent surgery is the only therapeutic intervention that has the potential to significantly prolong survival. However, only 20–30% of patients are potentially eligible for surgery at diagnosis due to the highly aggressive disease biology resulting in either locally advanced unresectable anatomy and/or metastatic presentation [1]. Management is further confounded because RECIST (Response Evaluation Criteria in Solid Tumors)-based treatment response assessment on conventional cross-sectional imaging (CT/MR) is suboptimal as the radiologic response often does not correlate with subsequent outcomes after neoadjuvant therapy (NAT). Recently, metabolic imaging with ^18^fluorodeoxyglucose ([^18^F]FDG), especially with PET/MRI, has been shown to improve staging, treatment response assessment, and prognostication of PDAC [2,3]. However, [^18^F]FDG is not specific for PDAC, and the performance of [^18^F]FDG PET for metastases is not high enough to supplant other invasive staging exams such diagnostic laparoscopy [4,5]. Therefore, there is an urgent need for novel diagnostic and staging tools to address unmet clinical needs and to improve the outcomes of patients with PDAC.

One critical factor that contributes to the dismal outcomes in PDAC is its characteristic intensely desmoplastic stroma, which typically constitutes 60–70% of its volume [6–8]. One of the most important stromal constituents are the cancer-associated fibroblasts (CAFs), which play a central role as drivers of fibrosis, immune escape, tumor cell dissemination, and treatment resistance [9,10]. Given their many tumor-promoting functions, CAFs are also a promising therapeutic target. Unlike fibroblasts in normal tissues, CAFs highly express a membrane-anchored peptidase, fibroblast activation protein (FAP), which is an independent predictor of poor outcomes in PDAC with almost linear correlation between the intensity of FAP expression and survival outcomes [11–14]. However, there has been very limited translation of these biological insights to clinical practice due to the lack of a noninvasive *in vivo* imaging strategy for FAP. Therefore, there is a critical unmet need to establish an imaging assay of FAP expression to advance our understanding of CAFs and to maximize their potential as diagnostic, prognostic, and therapeutic targets in clinical oncology.

Recently, quinolone-based compounds with a DOTA-chelator moiety have been developed for FAP-targeted imaging and theranostics [15]. Results of PET imaging with Fibroblast Activation Protein Inhibitors (FAPI) family of compounds have been published in more than 5000 patients without serious adverse events [16]. One of the FAP-targeted radiotracers, gallium-68 [^68^Ga]FAPI-46, has many favorable properties—low nanomolar affinity to FAP, near-complete internalization of radioactivity bound to FAP, favorable dosimetry and diagnostic isotope labeling profile, rapid blood clearance, prolonged retention and minimal physiologic uptake resulting in high image contrast [15]. These kinetics are also ideal for therapeutic applications and facilitate tagging of common therapeutic radioisotopes [e.g. the β emitter Lutetium-177 (^177^Lu)] to FAPI for radioligand therapy (RLT). The purpose of this multi-center, single arm, open label, non-randomized study (clinicaltrials.gov identifier NCT05262855) [17] is to prospectively evaluate the diagnostic performance of [^68^Ga]FAPI-46 PET for the detection of CAFs with FAP expression in patients with resectable or borderline resectable PDAC.

## Methods

### Study objectives and endpoints

#### Objectives

**Primary Objective**

- Evaluate the performance [sensitivity, specificity, accuracy] of [^68^Ga]FAPI-46 PET to detect FAP-expressing cells using histopathology as truth standard.

**Secondary Objectives**

- Evaluate positive and negative predictive values of [^68^Ga]FAPI-46 PET to detect FAP-expressing cells using histopathology as truth standard.
- Correlate the histopathology with FAP staining on FAP IHC assay.
- Further validate the safety profile of [^68^Ga]FAPI-46 in patients with PDAC

**Exploratory Objectives**

- Compare the detection of metastatic disease using [^68^Ga]FAPI-46 PET to a composite of clinical, radiological (i.e., CT, MR), and histopathological reference.
- Compare pre- and post-treatment [^68^Ga]FAPI-46 PET in Cohort 2 to identify perturbations, if any, from neoadjuvant therapy.

#### Endpoints

**Primary Efficacy Endpoint**

- Sensitivity, specificity, and accuracy will be determined on a per-lesion basis for all lesions with tissue available for analysis.
- Lesions positive for FAP as detected by [^**68**^Ga]FAPI-46 PET imaging.
- Malignant lesions as detected by histopathology.

**Secondary Efficacy Endpoints**

- Positive and negative predictive value will be determined on a per-lesion basis for all lesions with tissue available for analysis.
- Lesions positive for FAP as detected by [^**68**^Ga]FAPI-46 PET imaging.
- Malignant lesions as detected by histopathology.
- Staining intensity assessed by IHC, using H-score, correlated to the uptake of [^**68**^Ga]FAPI-46, using SUV_**max**_.
- Lesions positive for FAP as detected by IHC.

**Exploratory Efficacy Endpoints**

- Number of metastatic lesions identified through cross-sectional imaging (i.e., CT, MR) and/or [^**18**^F]FDG PET
- Number of [^**68**^Ga]FAPI-46 identified metastatic lesions.
- For cohort 2, change in radiotracer uptake using SUV_**max**_ percentage change in [^**68**^Ga]FAPI-46 between pre- and post-neoadjuvant therapy.

**Safety endpoints**

- Incidence and severity of treatment-emergent adverse events (TEAEs) or treatment-emergent serious adverse events (TESAEs) 24 hours from [^**68**^Ga]FAPI-46 administration according to MedDRA/CTCAE V.5.0.

### Study design

**Fig 1:**
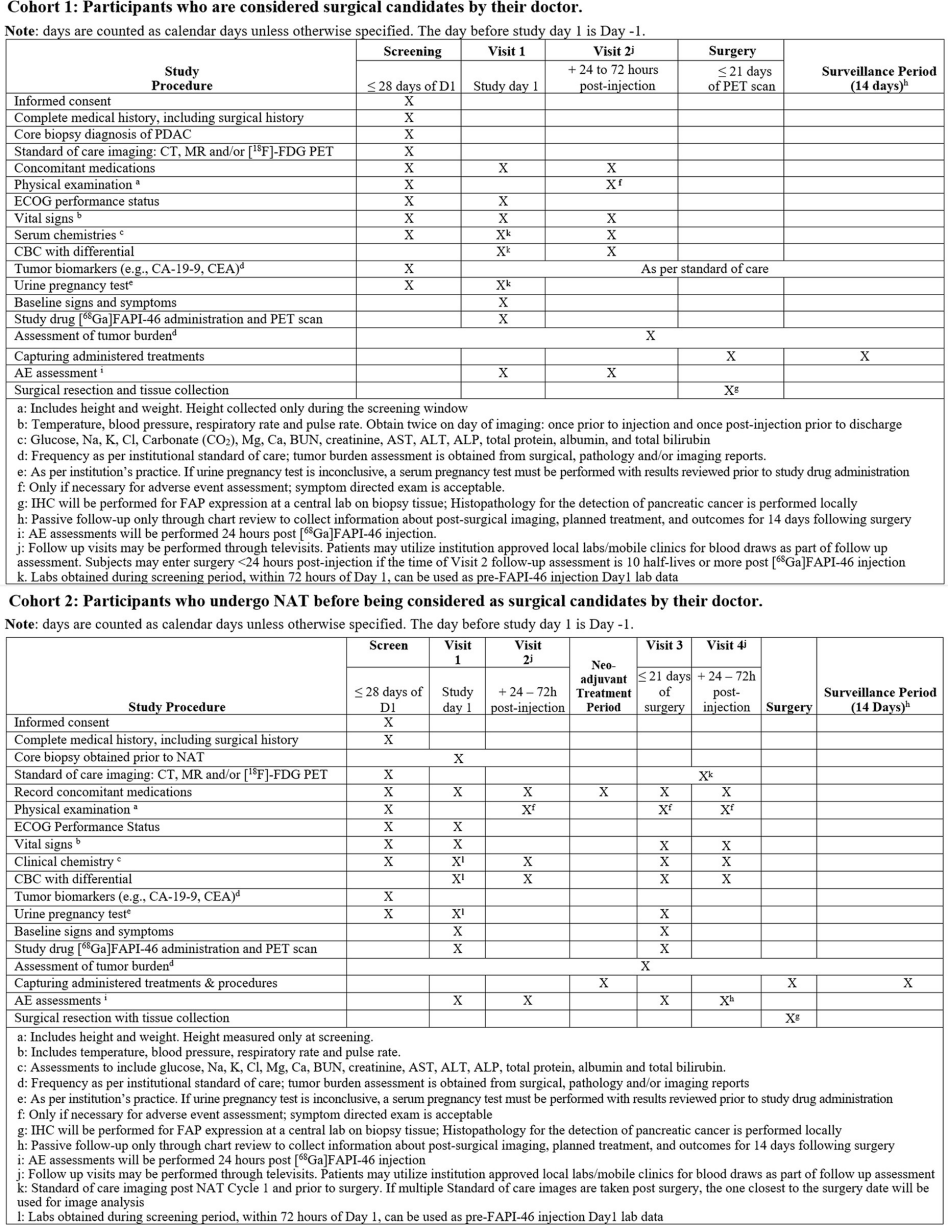
Schedule of activities.

**Fig 2:**
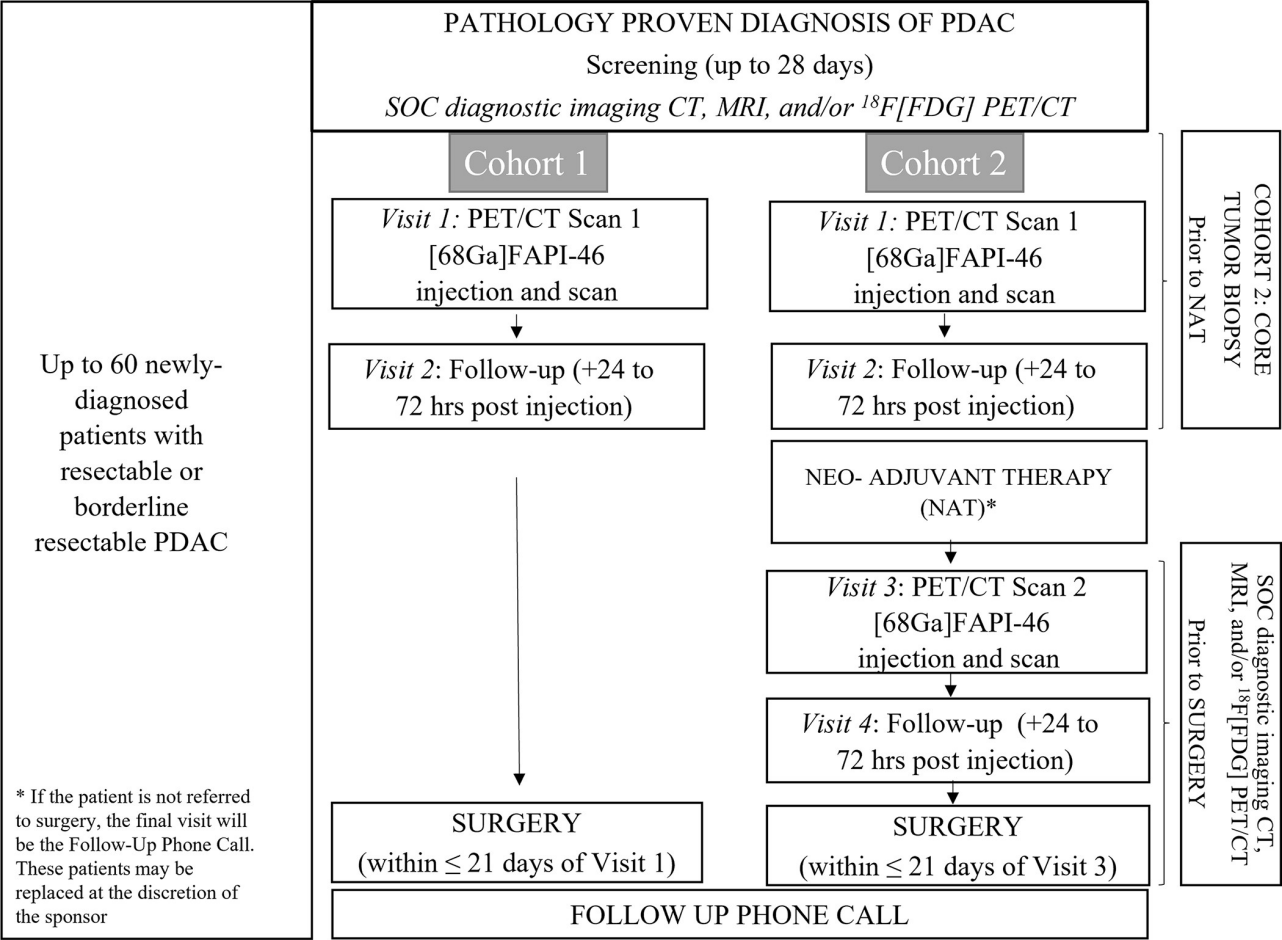
Study schematic.

Up to 60 adult treatment-naïve patients with pathologically confirmed PDAC who are candidates for either upfront curative-intent surgical resection (Cohort 1) or for surgical resection following neoadjuvant treatment (NAT) (Cohort 2) will be enrolled after a written informed consent is obtained by designated site-specific study coordinator (Figs 1 and 2). Each of the sites–Mayo Clinic (Rochester, Minnesota, USA), University of California Health (Los Angeles, California, USA), New York University Medical Center (New York, New York, USA), and BAMF Health (Grand Rapids, Michigan, USA)—has obtained approval of the study protocol from their respective Institutional Review Boards (IRB)—Mayo Clinic IRB (ID: 22–003295; approval date: 8/5/2022), University of California Los Angeles IRB (ID: 22–000631; approval date: 8/8/2022), New York University Langone IRB (ID: i21-01680; 4/29/2022) and WIRB Copernicus Group (WCG) IRB (ID: 20230369; approval date: 2/28/2023). The study was activated in May 2022. The study is under active enrollment with around 45% of target enrollment completed as of September 2023. The study is anticipated to be completed by May 2024.

**Inclusion criteria**

1. Patients with pathologically confirmed pancreatic ductal adenocarcinoma.
2. Treatment-naïve
3. Staged as anatomically resectable or borderline-resectable.
4. Planned to undergo surgical resection or to receive NAT (i.e., chemotherapy, radiation therapy, or combination) and subsequent planned surgical resection.
5. Anatomic imaging (e.g., CT, MRI) obtained within ≤ 28 days of consent.
6. Age ≥ 18 years

**Exclusion criteria**

1. Pregnant as determined by a pregnancy test as per institutional guidelines for individuals of child-bearing potential.
2. Declining to use effective contraceptive methods during the study (for individuals of child-producing potential)
3. Need for emergent surgery that would be delayed by participation.
4. Bacterial, viral, or fungal infections requiring systemic therapy, that are expected to impact FAP expression.
5. Serious co-morbidities and serious nonmalignant disease (e.g., hydronephrosis, kidney failure, liver failure, systemic or local inflammatory or autoimmune diseases or other conditions) that could compromise patient safety and/or protocol objectives.
6. Known diagnosis of an autoimmune disorder, that is expected to impact FAP expression.
7. Patients receiving any other investigational agent within the past 28 days.
8. Breastfeeding. However, nursing mothers are allowed if the potential participant commits to pumping breast milk and discarding it from injection to ≥ 24 hours from the time of the [^68^Ga]FAPI-46 injection.
9. Known hypersensitivity to any excipients used in [^68^Ga]FAPI-46, which include sodium acetate sodium ascorbate and/or hydrochloric acid.

#### Concomitant medication

No other investigational medical products are allowed during this study, from eligibility determination to surgery. Participant may receive investigational medical products post-surgery for the purpose of another study. Drugs used off-label consistent with institutional practice or the standard-of-care do not meet the definition of investigational medical product. Participants would receive full concomitant care during this study consistent with institutional practices and standard-of-care.

#### Follow-up

Passive follow-up only through chart review to collect information about post-surgical imaging, planned treatment, and outcomes for 14-days following surgery.

### Study intervention and procedure

An overview of the study is provided in Fig 1. The timing of each visit is relative to prior visit. The [^68^Ga]FAPI-46 PET scans will be acquired after initial staging is done using institutional standard methods. For patients in cohort 2, a second [^68^Ga]FAPI-46 PET scan will be performed within 21 days prior to planned surgical resection. This will be followed by histopathology and IHC analyses of resected PDAC tumor specimens. The surgeon will not be blinded to the [^68^Ga]FAPI-46 scans prior to surgery. Participants who withdraw, are withdrawn, do not complete the [^68^Ga]FAPI-46 PET scan ≤ 21 days prior to surgery (Cohort 2), do not have a core biopsy prior to NAT, or who are not surgical candidates post-NAT may be replaced

#### Synthesis of [^68^Ga]FAPI-46

**Fig 3:**
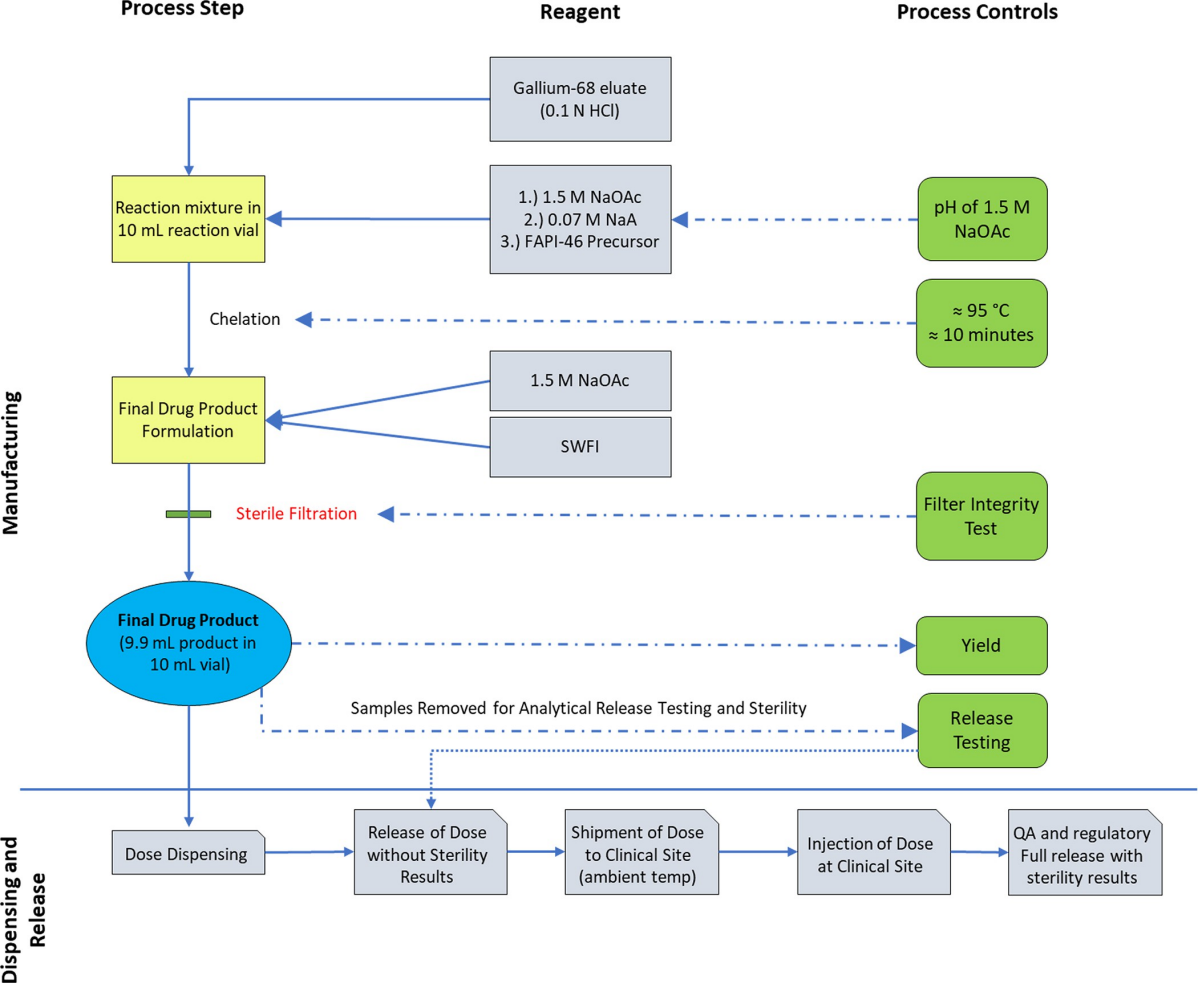
[^68^Ga]FAPI-46 drug product manufacturing process diagram.

FAPI-46 precursor is a small molecule critical drug intermediate consisting of a FAPI moiety conjugated with a DOTA chelator for use in manufacturing the radiolabeled final drug product. FAPI-46 precursor is supplied in single use vials (0.050 mg ± 20%) from ABX (Advanced Biochemical Compounds GmbH, Germany) in powder form. The radionuclide in this study, gallium-68, is a neutron deficient isotope (31 neutrons and 37 protons) of gallium, which decays via electron capture and positron emission to zinc-68. Gallium- 68 has a 68-minute half-life, with a relatively low translational energy of the positron (maximum energy of 1.9 MeV) for producing high quality PET images. FAPI-46 precursor is radiolabeled with gallium-68 to produce the final drug product [^68^Ga]FAPI-46. The resulting material contains buffering excipients that maintain the stability to produce the [^68^Ga]FAPI-46 final drug product (Fig 3).

#### Formulation, appearance, packaging, and labeling

The final [^68^Ga]FAPI-46 drug product is a clear, colorless, and particulate free, sterile solution in 10 mL Type 1 glass vial. The drug product contains 0.1 mCi/ml to 5.0 mCi/ml [^68^Ga]FAPI-46 at the end-of-synthesis, 11.81 mg/m sodium acetate and 0.49 mg/mL sodium ascorbate in sterile water for injection in approximately 9.9 ml.

#### Storage and stability

The drug product is stored upright in lead-shielded container at 15–25°C with use before expiration of 4 hours post end-of-synthesis. Handling of the investigational medical product will be as per the radioactive material regulations and licensing requirements.

#### Dosing, administration, and PET scan

**Fig 4:**
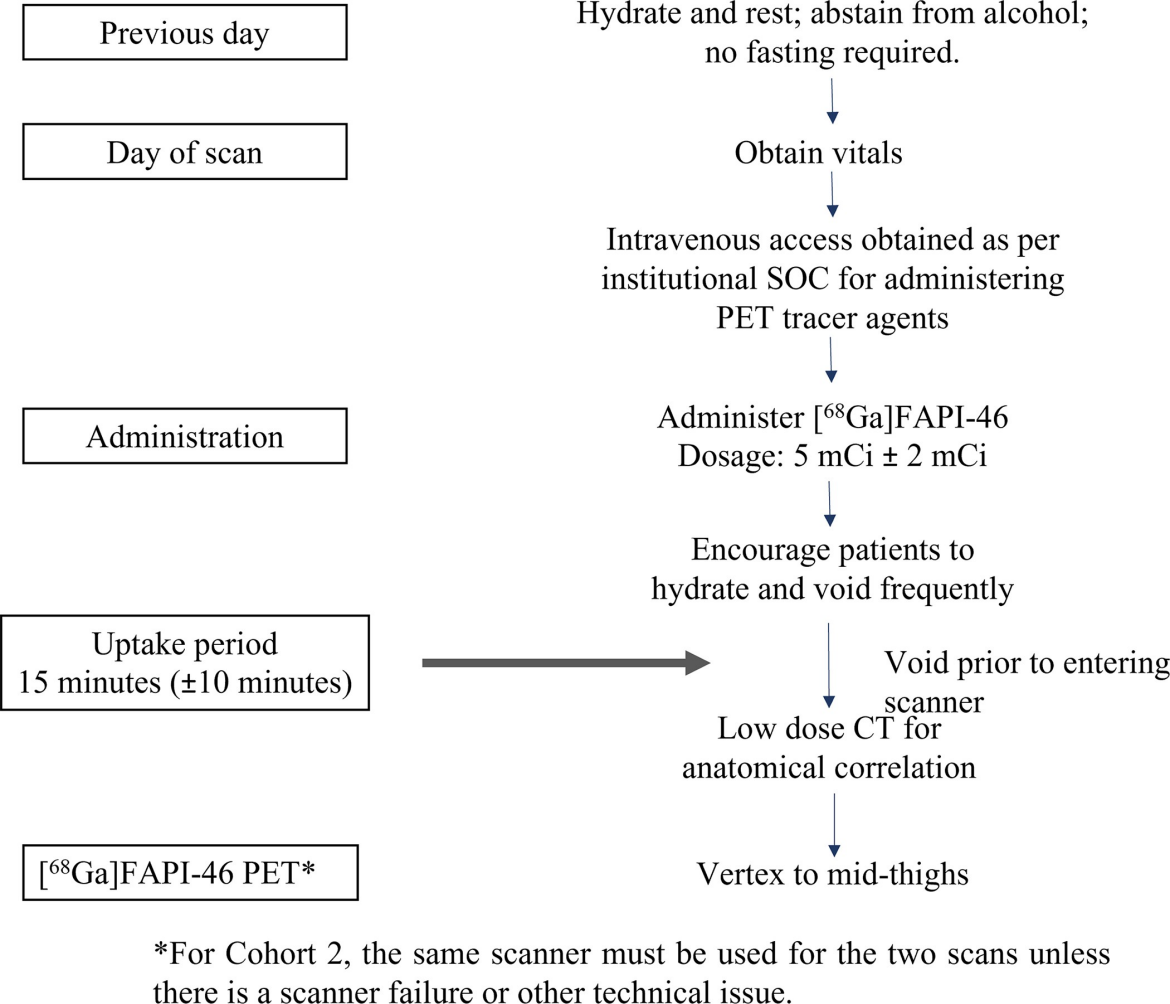
Summary of prior patient preparation and scanning procedure.

Participants will be directed to hydrate well the day prior to scan. The scan does not require fasting, but participants will be asked to abstain from alcohol one day prior to scan. Vital signs will be obtained prior to initiating procedures. Radiolabeled [^68^Ga]FAPI-46 (5 mCi ± 2 mCi) will be administered intravenously 15 minutes (±10 minutes) prior to the initiation of the attenuation correction CT as per institutional policies. During this uptake period of 15 minutes (±10), participants will be encouraged to hydrate and void. Participants will be directed to void prior to being placed on the scanner. Scanner coverage will be set up to extend from vertex to mid-thighs. Low dose CT will be used for anatomical correlation (Fig 4). For participants in Cohort 2, the same scanner will be used for the two scans unless there is a scanner failure or other technical issues. All interventions in this protocol will be administered by licensed medical staff under the supervision of the study investigator.

#### Tissue specimen collection and handling

All tissues collected at biopsy or surgery will be processed according to institutional standard for local histopathological assessment. FAP IHC will be conducted by a core laboratory (Discovery Life Sciences, Newtown, PA, USA). Detail procedures, scoring mechanism and validation results of FAP IHC are provided in Appendix 1 S4 File.

### Study assessment

#### Adverse events

The adverse event (AE) collection window is from the time of injection of [^***68***^Ga]FAPI-46 through 24 hours post-injection. If the 24-hour window would end on a holiday or non-clinic day (or the participant cannot return to clinic due to unforeseen circumstances), the window may be extended until 72 hours post-injection. Adverse event collection windows are tied to the number of [^***68***^Ga]FAPI-46 injections. Participants in Cohort 1 will have one AE collection window whereas those in Cohort 2 will have two discrete AE collection windows. This study defines AE terms as per the U.S. federal code at 21CFR§312.32. The severity of AE will be evaluated utilizing the CTCAE v.5 criteria. The relationship of [^***68***^Ga]FAPI-46 PET scan procedure to an adverse event will be assessed utilizing the FDA’s December 2012 guidance Safety Reporting Requirements for INDs and BA/BE Studies.

#### Clinical data

Data from electronic medical records from prior (pre-consent) and routine medical care visits, as well as data collected during study visits will be entered into an electronic data capture system.

#### Imaging data

Interpretation and analyses of the imaging data will be conducted by a central imaging core lab followed by delivery of the data to the sponsor. During the independent review, a single board-certified nuclear medicine physician will provide assessment of the [^68^Ga]FAPI-46 PET. The assessment will include [^68^Ga]FAPI-46 uptake metrics, including tumor-to-background (TBR) ratios of the primary tumor and positive local lymph nodes. The co-acquired attenuation correction CT will also be presented with the PET scan.

Cohort 1 and cohort 2 pre-NAT [^68^Ga]FAPI-46 PET scans acquired during the study will be presented in a blinded fashion and in a random order. Blinding includes the participant demographics, the identity of the sites and participants, treatment arm, and all other information not essential to the independent review. For the cohort 2 post NAT scan, the reader will compare the pre- and post-treatment scan, which will allow for assessment of the exploratory objective of the study in monitoring changes in [^68^Ga]FAPI-46 before and after NAT.

All PET/CT imaging will be viewed on dedicated PET/CT software, which allows for simultaneous display of the PET, fused PET/CT, and co-acquired attenuation correction CT. The reviewer will assess the image quality of each scan and record the result in the rules-based electronic case report form (eCRF). The eCRF will provide 3 general quality categories of *Optimal*, *Readable but Not Optimal*, and *Not Readable*. If the reader selects *Readable but not Optimal* or *Not Readable*, the reader will be required to document the reason in a free text field. The reviewer will then perform a systematic evaluation of the PET scan, first evaluating the image overall for biodistribution, artifacts, altered anatomy, etc. Next, the primary tumor (or tumors) will be identified and annotated. The reviewer will choose the SUV measuring tool that is optimal for the task and will likewise adjust the volume of interest (VOI) as necessary to define the boundary of tumor uptake on PET (while excluding any adjacent non-tumoral uptake). The software automatically generates SUV_max_ from the VOI. The reviewer will then evaluate local lymph nodes positive for radiotracer uptake and follow the same process of creating VOIs for the nodes as has been specified for the primary lesion.

Potential Metastases: [^68^Ga]FAPI-46 PET/CT will be analyzed for lesions that are visually considered as suggestive of metastases based on the morphology, focality and intensity of [^68^Ga]FAPI-46 uptake. Lesions will be counted, classified with respect to their locations (liver, peritoneum, non-regional lymph nodes, lung, bone, and other), and their [^68^Ga]FAPI-46 avidity will be semi-quantitatively analyzed through SUV_max_. Standard-of-care imaging will be evaluated with established clinical criteria for the assessment of metastases.

Reference tissues: The reviewer will also place 2-cm in diameter spherical VOIs on the following reference tissues (i.e., reference VOIs): liver, blood pool (lumen of the descending thoracic aorta), gluteal muscle, and normal pancreas, if possible. The system will calculate the SUV_max_ for each reference VOI.

#### Immunohistochemistry and histopathology data

**Fig 5:**
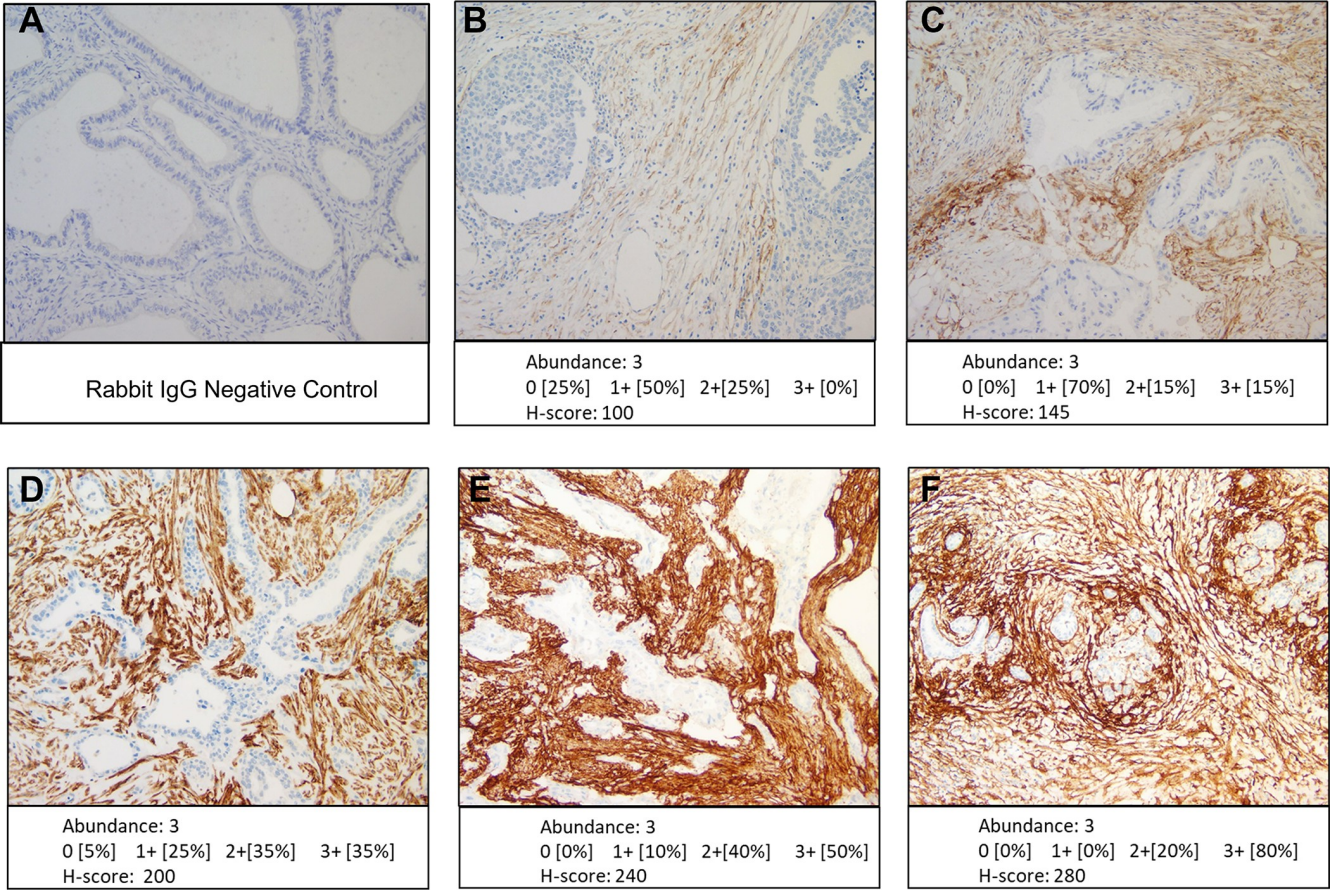
Fibroblast activation protein immunohistochemistry (FAP IHC). Representative micrographs of immunohistochemical FAPα staining in pancreatic cancer showing variations in levels of staining. A is the negative control rabbit IgG and B-F are stained with FAPα.

**Fig 6:**
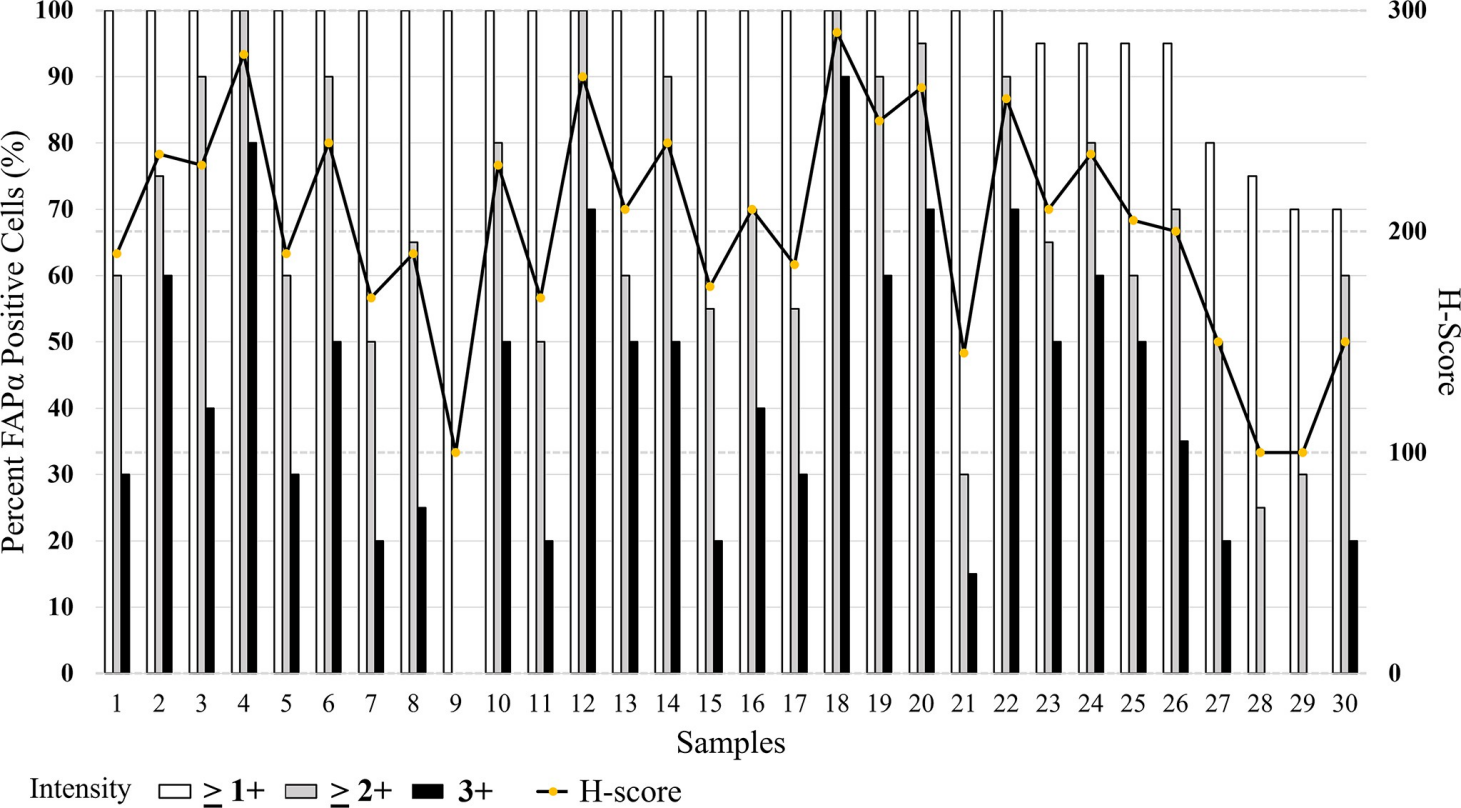
Percent score and H-score for 30 pancreatic cancer tissues. The left axis of the graph shows the scale (1–100%) correlating to the percentage FAPα positive cells. The percent scores for each sample are represented as a bar graph, the white boxes represent the percentage of cells that stained at an intensity of > 1+, the grey boxes represent the percentage of cells that stained at an intensity of >2+, and the black boxes represent the percentage of the cells that staining at an intensity of 3+. The right axis of the graph shows the scale (0–300) for the H-scores. The H-score is represented by the (yellow) dots.

In subjects, formalin-fixed, paraffin-embedded (FFPE) tissue sections from biopsied or resected specimens will be evaluated by standard histopathologic methods at an independent centralized core facility (Discovery Life Sciences, Newtown, PA, USA). FAP expression levels will be evaluated using a FAPα IHC assay, which was previously validated in a GCLP (Good Clinical Laboratory Practice) facility (Discovery Life Sciences, Newtown, PA, USA) (Figs 5 and 6). As part of the study, a histopathology assessment will be performed to indicate presence or absence of tumor tissue being analyzed. In addition, FAP IHC scoring will be performed by providing percent (%) tumor and a percent (%) stroma score, along with semiquantitative analysis of the abundance of stromal staining as 0, 1, 2, and 3, followed by the H-score as described in Appendix 1 in S4 File.

### Statistical analysis and justification of sample size

The study population for all analyses will be defined as all patients enrolled in the study who receive at least one dose of [^68^Ga]FAPI-46. Patients who exit the study prior to receiving study medication may be replaced. For this study, the primary efficacy endpoints are sensitivity and specificity, each of which will be evaluated with a hypothesis test:

- H0: Sensitivity (Specificity) ≤ Performance Goal (PG)
- H1: Sensitivity (Specificity) > Performance Goal (PG)

Success is defined as rejecting the null hypothesis for the sensitivity hypothesis tests. The specificity test will have lower power given the lower number of samples that are likely to be negative from the histopathology sample. The minimum sample size necessary to achieve desired power for sensitivity was computed for the hypothesis test above utilizing a one-sample binomial proportion test with a normal approximation, an upper one-sided significance level of 0.05, and the following parametric assumptions:

- Performance Goal (PG) = 0.75 for sensitivity and 0.70 for specificity
- True Sensitivity and Specificity = 0.90

Given the above parametric assumptions, 60 subjects will yield 92.7% power to demonstrate that the true sensitivity and specificity is greater than 0.75.

#### Safety analysis

All patients who receive any amount of [^68^Ga]FAPI-46 will be included in the final summaries and listings of safety data. Frequencies of patients experiencing at least one AE will be displayed by body system and preferred term according to MedDRA terminology. Intensity (severity) of the AEs will be graded according to the CTCAE v4.0.

Summary tables will present the number of patients observed with AEs and corresponding percentages. The denominator used to calculate incidence percentages will consist of patients receiving at least one dose of study medication. Within each table, the AEs will be categorized by MedDRA body system and preferred term. Additional subcategories will be based on event intensity and relationship to study drug. Deaths and other SAEs will be tabulated. Vital signs will be summarized using descriptive statistics. Summary tables will be prepared to examine the distribution of laboratory measures over time.

#### Efficacy analysis

*Primary efficacy analyses*. The primary efficacy endpoints will compare the results from [^**68**^Ga]FAPI-46 PET and histopathology. The primary analysis of sensitivity and specificity will be summarized using frequency counts and percentages as well as 95% asymptotic normal confidence intervals. Inference for the primary hypotheses will be conducted utilizing a one-sample binomial proportion test with a normal approximation, a one-sided upper significance level of 0.05, and assumed null proportions/performance goals of 0.75 for sensitivity. Success for the primary efficacy analyses will be defined as sensitivity that is statistically significantly greater than the performance goal. Accuracy of [^**68**^Ga]FAPI-46 PET for the detection of FAP expressing cells compared to histopathology will be presented with a 95% confidence interval.

*Secondary efficacy analyses*. Association between SUV_max_ from [^68^Ga]FAPI-46 PET on cross sectional imaging and FAP expression from IHC will be summarized by correlation coefficients.

#### Access to data and materials

The lead academic investigator and other site leads will have full access to all interim and final data results of the study. All data generated and/or analyzed during this study will be available in a future publication.

#### Dissemination of results

The results of this trial will be submitted for publication in national or international peer-reviewed journals with all collaborators acknowledged. Results will also be disseminated through conference presentations. It is expected that several publications will originate from this protocol, addressing the aims as mentioned above.

## Discussion

PDAC is an aggressive malignancy with a dismal 5-year survival rate due to its propensity for widespread metastatic dissemination at the time of initial diagnosis. CAFs are one of the most important stromal constituents in PDAC. Besides being the principal source of extensive extracellular matrix, CAFs play pleiotropic roles in the biology of PDAC [9,10]. Gaining deeper insights into their role is essential to overcome therapy resistance, develop novel targeted therapies, and improve patient outcomes. During their activation, CAFs undergo morphological changes and express specific surface markers, with FAP being highly and consistently upregulated [11–14]. Currently, FAP expression can only be measured *in vitro* through methods such as immunohistochemistry (IHC), mRNA or protein analysis using samples obtained invasively through biopsy or surgery. These methods allow for analyses only at a single time point. Moreover, tissue sampling fails to capture the inherent heterogeneity in FAP expression observed within and among lesions. Existing options also fail to characterize the phenotypic plasticity and functional diversity of different subsets of CAFs [10]. Consequently, there is a crucial unmet need for a non-invasive imaging technique for FAP-expressing CAFs.

Small and retrospective studies have shown strong potential of FAPI PET in PDAC [18–22]. For instance, in patients with primary and recurrent PDAC, [^68^Ga]FAPI-04 PET/CT was seen to change disease stage and subsequent therapeutic management. Additionally, there was a linear correlation between FAP expression in the tumor stroma and [^68^Ga]FAPI-04 PET uptake [22]. When compared against [^18^F]FDG PET/CT, [^68^Ga]FAPI PET/CT was seen to have higher sensitivity for assessing primary tumors, lymph nodes, and distant metastases, which led to upgrades in disease stage and changes in therapeutic strategy. FAPI PET has shown potential to improve the consistency and precision in radiation target volume delineation for PDAC compared to CT [21], which is important in view of high variability in PDAC segmentation on CT due to its infiltrative growth pattern [23–25]. FAPI PET also represents an attractive prospect to guide innovative radiation dose painting approaches since there is no physiologic uptake of FAPI in sub-adjacent radiation-sensitive organs such as small bowel. In summary, the available data provide strong rationale for its systematic and prospective evaluation in PDAC.

Generally, the benchmarks to meet FDA approval criteria for PET radiotracers are more favorable than for therapeutic drugs. As an example, FDA does not require clinical outcome data for potential approval of PET radiotracers. Yet, only a few PET radiotracers are approved by FDA, averaging about one every two years. Therefore, to realize the promising potential of FAPI PET in PDAC and to maximize the likelihood of meeting FDA standards, it is necessary to execute a coherent prospective study. With that intent, the central hypothesis of the current study is that [^68^Ga]FAPI-46 PET will be an accurate technique to detect and quantify FAP-expressing CAFs in PDAC. To test the hypothesis, we will characterize the immunohistochemical expression of FAP in tumor samples using a previously validated IHC assay and compare it against tumor uptake of [^68^Ga]FAPI-46 on PET.

A significant challenge arises in potentially resectable PDAC cases, as critical locoregional vasculature involvement often renders surgery infeasible unless tumors respond to NAT. NAT improves outcomes by increasing the likelihood of achieving negative margin resection, an independent predictor of survival, and by targeting occult metastases commonly present in patients regardless of initial radiologic staging exams. Interestingly, despite having more advanced tumors, patients who complete NAT and undergo margin-negative resection with major pathologic response demonstrate superior outcomes compared to those who undergo upfront resection. Therefore, NAT is emerging as the new treatment standard given the high probability of occult metastases in most patients and the high rates of metastatic recurrence after seemingly curative local resection [26–32]. To accommodate the evolving practice patterns, our study incorporates several measures. Firstly, IHC will be conducted on available initial diagnostic biopsy samples, and the results will be correlated with baseline FAPI PET findings. Secondly, the expected intricate changes in FAP expression from NAT will be equally captured by both post-NAT FAPI PET and IHC. Consequently, evaluating the performance of FAPI PET against IHC and histopathology on surgical specimens is anticipated to be feasible without significant challenges, aligning with our research objective. Finally, our cohort 1 comprises patients who will undergo upfront resection. Within this group, we will compare the FAP expression profile on surgical specimens obtained from treatment-naïve PDAC with the uptake of [^68^Ga]FAPI-46 observed on PET. These study design considerations were discussed during our pre-Investigational New Drug (pre-IND) meetings with the FDA and reflected in the clinical protocol approved for this study.

The current study also encompasses a head-to-head comparison between ^68^Ga-FAPI-46 PET and standard-of-care imaging modalities (e.g., [^18^F]FDG PET and CT). FAPI PET offers a complementary approach to [^18^F]FDG PET by utilizing a different uptake mechanism. [^18^F]FDG uptake is dependent upon glucose transporter and hexokinase activity, and the number of tumor cells with high glycolytic activity. Conversely, FAPI uptake is dependent upon the CAF-containing stroma volume and the level of FAP expression. Given the larger stroma volume in PDAC compared to epithelial cell volume [8], FAPI PET holds the potential for enhanced sensitivity in lesion detection compared to glucose metabolic PET. Additionally, FAPI PET exhibits high image contrast as normal organs like the liver and bowel do not show physiological FAPI uptake. These imaging characteristics contribute to improved sensitivity in detecting metastases in common sites such as the liver and peritoneum [19,20,22]. The current study will evaluate the performance of standard-of-care imaging in detecting metastases and compare it to [^68^Ga]FAPI-46 PET. Based on these distinctive features and prior research, we posit that [^68^Ga]FAPI-46 PET will provide incremental information for accurate pre-surgical staging.

The evaluation of ^68^Ga-FAPI-46 PET in PDAC is also a critical pre-requisite for the exciting prospect of FAPI-based radioligand therapy (RLT) as a novel therapeutic option in PDAC. RLT entails tagging ligands such as FAPI-46 with radioisotopes that emit therapeutic radiation (e.g.,^177^Lu or ^90^Y). Such agents tend to deposit directly in tumors with minimal-to-no side effects on normal tissues. RLT can deliver ionizing radiation to CAFs directly and to PDAC cells via crossfire effects. Combination of α and β particles can further improve these dual anti-tumor effects via short-range α-radiation to CAFs and mid-to-long-range β-radiation to neoplastic cells. Promising results from pre-clinical studies [33,34] have paved the way for clinical trials of such RLT strategies [35]. Targeting FAP to deplete stromal CAFs may also disrupt cancer supportive functions and inhibit PDAC growth. Furthermore, by breaking the stroma barrier, the effects of other pharmacologic, immunologic, radiation- or cell-based therapies can be potentiated [36,37]. Thus, outcome of the current study will have significant implications for the theranostic paradigm in patients with PDAC.

In conclusion, there is a critical need for a non-invasive imaging technique to assess FAP-expressing CAFs in PDAC. Currently, FAP expression can only be measured through invasive methods, limiting the understanding of CAF heterogeneity and functional diversity within and among lesions. The strong potential of FAPI PET in PDAC has been demonstrated in small retrospective studies. The current study aims to systematically evaluate the performance of [^68^Ga]FAPI-46 PET by comparing it to IHC and standard-of-care imaging modalities. Additionally, the evaluation of FAPI PET is a crucial component for the development of FAPI-based RLT, which offers targeted radiation delivery to CAFs and PDAC cells, disrupting cancer-supportive functions and potentiating other therapeutic approaches. The outcomes of this prospective, multi-center study could have significant implications for improving patient outcomes and advancing the theranostic paradigm in PDAC. The clinical evidence from this work could form the basis of a new drug application (NDA) with the FDA.

## Supporting information

S1 Checklist SPIRIT 2013 checklist: Recommended items to address in a clinical trial protocol and related documents*. (PDF)

S1 File Study protocol. (PDF)

S2 File Letter confirming funding. (PDF)

S3 File IRB/Ethics committee approval letter for each of the sites. (PDF)

S4 File (DOCX)
