# Supplementary material for: Gallium-68-labeled fibroblast activation protein inhibitor-46 PET in patients with resectable or borderline resectable pancreatic ductal adenocarcinoma: A phase 2, multicenter, single arm, open label non-randomized study protocol
Source: PLoS One. 2023 Nov 27;18(11):e0294564. doi: 10.1371/journal.pone.0294564 (PMC10681241; doi:10.1371/journal.pone.0294564)
Supplement: S1 File — (PDF) [file pone.0294564.s002.pdf]

CLINICAL STUDY PROTOCOL

**Study Title:**	A Phase 2, Multicenter, Single Arm, Open Label Non-Randomized Study of [^{68}Ga]FAPI-46 PET in Patients with Resectable or Borderline Resectable Pancreatic Ductal Carcinoma
**Investigational Product**:	[^{68}Ga]FAPI-46
**Sponsor**:	Sofie Biosciences, Inc<br>21000 Atlantic Boulevard<br>Suite 730<br>Dulles, VA 20166
**IND No.:**	151901
**Protocol Number**:	GaFAPI-2022P2
**Protocol Version/Date:**	Version 5.0. 31st, March 2023

This protocol was designed and will be conducted, recorded, and reported in compliance with the principles of Good Clinical Practice (GCP) guidelines, as adopted by U.S. Federal Law.

1. PROTOCOL SUMMARY

1.1. Synopsis

**Study Title:**	A Phase 2, Multicenter, Single Arm, Open Label, Non-Randomized Study of [^{68}Ga]FAPI-46 PET in Patients with Resectable or Borderline Resectable Pancreatic Ductal Carcinoma
**Sponsor**	Sofie Biosciences, Inc<br>21000 Atlantic Boulevard<br>Suite 730<br>Dulles, VA 20166
**Protocol Number**<br>**Version:**<br>**Date**	GaFAPI-2022P2<br>V5.0<br>31st, March 2023
**Study Description:**	Prospective, multi-center, open label, non-randomized clinical trial to assess efficacy of [^{68}Ga]FAPI-46 to detect FAP expressing cells in patients with resectable or borderline resectable PDAC. The [^{68}Ga]FAPI-46 PET scan will be acquired in patients with pathologically proven PDAC after initial staging using institutional standard methods and prior to surgical resection. The PET scan results will be compared to histopathology and IHC analyses (truth standard) of the resected PDAC tumor specimens.
**Study Rationale**	Positron Emission Tomography (PET) is a 3D molecular imaging modality that utilizes radiolabeled molecules in trace amounts (~10-12 to 10-15 moles/g) ("tracers") to target and measure biological and pathophysiological processes. Researchers often validate these probes [molecules] to examine microorganisms, cells, and preclinical animal models then advance to patients further confirming efficacy and safety to provide visualization and characterization of the biology and biochemistry of health and disease. In oncology, PET modality has been extensively validated, proven, and well accepted for diagnosis, staging, restaging and also, evaluating therapeutic efficacy, establishing it as a key technology for personalized, precision medicine.<br>Fibroblast activation protein (FAP) is a type II transmembrane glycoprotein with unique expression profile in multiple malignancies. It has low to absent expression in healthy tissues but varying degrees of expression in wound healing, inflammation, fibrosis, and cancer-associated fibroblasts (CAFs). FAPI-46 is one of the validated inhibitor small molecules that has high binding properties to FAP which renders it an important diagnostic marker in visualizing the tumor and the stroma and thereof, the microenvironment (TME). Besides the clear diagnostic potential based on FAP expressing cells, FAP may serve as therapeutic target to harness the ECM (extracellular matrix), alone or in combination with other therapies that directly target the tumor, including chemo-, immunologic, radiation-, or cell-based therapies whose function may be otherwise blunted or blocked by the tumor stroma. An effective marker of FAP expressing cells would provide significant value in identifying patients who might benefit from FAP therapeutic applications in addition to potentially monitoring response to non-FAP therapies.<br>[^{68}Ga]FAPI-46 is a radiopharmaceutical indicated for PET imaging of FAP-expressing cells.

**Objectives:**	Primary Objective:<br>1. Evaluate the performance [sensitivity, specificity, accuracy] of [^{68}Ga]FAPI-46 PET imaging to detect FAP-expressing cells, using histopathology as truth standard.<br><br>Secondary Objectives:<br>1. Evaluate positive and negative predictive values of [^{68}Ga]FAPI-46 PET images, to detect FAP-expressing cells using histopathology as truth standard.<br>2. Correlate histopathology with FAP staining on FAP IHC assay.<br>3. Further characterize the safety profile of [^{68}Ga]FAPI-46 in patients with PDAC.<br><br>Exploratory Objectives<br>1. Compare the detection of local and metastatic disease using [68Ga]FAPI-46 PET to a composite of clinical, radiological (i.e. CT, MR) and/or 18F-FDG PET and histopathological reference in patients with resectable or borderline resectable PDAC.<br>2. Compare pre- and post-treatment [^{68}Ga]FAPI-46 PET evaluations obtained in Cohort 2 to identify perturbations, if any, from neoadjuvant therapy.
**Planned Total Sample Size**	Up to 60 patients with newly diagnosed resectable or borderline resectable PDAC will be enrolled.
**Primary Inclusion Criteria**	1. Pathologically confirmed pancreatic ductal adenocarcinoma<br>2. Treatment-naïve<br>3. Staged as resectable or borderline-resectable<br>4. Planned to undergo surgical resection or to receive neoadjuvant therapy (i.e., chemotherapy, radiation therapy, or combination) and subsequent possible surgical resection<br>5. Anatomic imaging (e.g., CT, MRI) obtained within ≤ 28 days of consent<br>6. Age ≥ 18 years<br>7. Completed informed consent as determined per the IRB of record
**Primary Exclusion Criteria**	1. Pregnant as determined by a pregnancy test as per institutional guidelines for individuals of child-bearing potential<br>2. Declining to use effective contraceptive methods during the study (for individuals of child-producing potential)<br>3. Need for emergent surgery that would be delayed by participation<br>4. Bacterial, viral, or fungal infections requiring systemic therapy, that are expected to impact FAP expression in the opinion of the sponsor or their designee<br>5. Serious co-morbidities and serious nonmalignant disease (e.g., hydronephrosis, kidney failure, liver failure, systemic or local inflammatory or autoimmune diseases or other conditions) that in the opinion of the investigator, physician of record and/or Sofie could compromise patient safety and/or protocol objectives.<br>6. Known diagnosis of an autoimmune disorder that is expected to impact FAP expression in the opinion of the sponsor or their designee<br>7. Patients receiving any other investigational agent within the past 28 days<br>8. Breastfeeding. Note: nursing parents are allowed if the potential participant commits to pumping breast milk and discarding it from injection to ≥ 24 hours from the time of the [^{68}Ga]FAPI-46 injection.<br>9. Known hypersensitivity to any excipients used in [^{68}Ga]FAPI-46: trace amounts of sodium acetate sodium ascorbate and/or hydrochloric acid
**Test Article Administration and Imaging**	Intravenous administration of radiolabeled [^{68}Ga]FAPI-46 (5 mCi ± 2 mCi) as per institutional policies, administered 15minutes (±10minutes) prior to the initiation of the attenuation correction CT followed by the PET acquisition encompassing the participant's vertex through mid-thighs. Up to two [^{68}Ga]FAPI-46 PET/CT scans may be performed per participant, depending on cohort.

**Duration of Participation**	**Unless otherwise specified, 'days,' refers to calendar days.** Study day 1 refers to the day the (first) [^{68}Ga]FAPI-46 PET occurs, day 2 is the following calendar day, etc.<br><br><u>Cohort 1</u><br>Participants receiving surgical intervention, not prescribed neoadjuvant therapy by their oncology team:<br>• Screening period (≤ 28 days from study day 1)<br>• Standard of care diagnostic modality: MR, CT and/or [^{18}F]-FDG PET<br>• Day 1: [^{68}Ga]FAPI-46 PET radiopharmaceutical administration, scan and safety assessment<br>• +24 to 72 hours post-injection of [^{68}Ga]FAPI-46: safety follow up assessment<br>• Surgical intervention ≤21 days of [^{68}Ga]FAPI-46 PET scan<br>• Tissue samples from surgical intervention assessed for FAP expression<br><u>Cohort 2</u><br>Participants who are candidates to receive surgical intervention, prescribed neoadjuvant therapy by their oncology team prior to surgery<br>• Screening period (≤ 28 days from study day 1)<br>• Standard of care diagnostic modality: MRI, CT and/or [^{18}F]-FDG PET<br>• Day 1: [^{68}Ga]FAPI-46 PET radiopharmaceutical administration, scan 1 and safety assessment<br>• +24 to 72 hours post-injection of [^{68}Ga]FAPI-46: safety follow up assessment<br>• Neoadjuvant therapy as prescribed by the oncology team. Core biopsy must be obtained prior to initiation of neoadjuvant therapy<br>• ≤ 21 days of surgery: [^{68}Ga]FAPI-46, radiopharmaceutical administration, scan 2 and safety assessment<br>• +24 to 72 hours post-injection of [^{68}Ga]FAPI-46: safety follow up assessment<br>• ≤ 21 days of surgery: Standard of care diagnostic modality: MR, CT and/or [^{18}F]-FDG PET<br>• Surgical intervention as per institutional standard and oncology team direction<br><br>Tissue samples from biopsy and surgical intervention assessed for FAP expression
**Data Evaluation**	<u>Clinical Data</u><br>Data from electronic medical records from prior (pre-consent) and routine medical care visits, as well as data collected during study visits will be entered into an electronic data capture system for analysis.<br><br><u>Imaging Data</u><br>Imaging data reads will be conducted by a central image reading lab followed by quantitative recording of the data.<br>Special Considerations: Potential Metastases: [^{68}Ga]FAPI-46 PET/CT will be analyzed for lesions that are visually considered as suggestive of metastases based on the morphology, focality and intensity of [^{68}Ga]FAPI-46 uptake. Lesions will be counted, classified with respect to their locations (liver, peritoneum, non-regional lymph nodes, lung, bone, and other), and their [^{68}Ga]FAPI-46 avidity will be semi- quantitatively analyzed through SUV_{max}. Standard-of-care imaging will be evaluated with established clinical criteria for the assessment of metastases.<br><br><u>Immunohistochemistry and Histopathology Data:</u><br>In patients who undergo surgery following completion of treatment as a part of standard clinical care, formalin-fixed, paraffin-embedded (FFPE) tissue sections from biopsied or resected specimens will be evaluated by standard histopathologic methods for FAP expression levels.

Statistical Analysis Plan and Justification of Sample Size

The sample size will be 60 patients in order to adequately power the primary objective of sensitivity.

For this study, the primary efficacy endpoints are sensitivity and specificity, each of which will be evaluated with a hypothesis test:

H0: Sensitivity (Specificity) ≤ Performance Goal (PG)
H1: Sensitivity (Specificity) > Performance Goal (PG)

Since it is known that most of the samples will be positive via histopathology, the sample size will be based on powering the test of sensitivity, as the number of samples for testing specificity are expected to be small.

The minimum sample size necessary to achieve desired power for sensitivity was computed for the hypothesis test above utilizing a one- sample binomial proportion test with a normal approximation, an upper one-sided significance level of 0.05, and the following parametric assumptions:

- Performance Goal (PG) = 0.75 for sensitivity and 0.70 for specificity
- True Sensitivity and Specificity = 0.90

Given the parametric assumptions above, 60 subjects will yield 92.7% power to demonstrate that the true sensitivity is greater than 0.75.

<u>Primary Efficacy Analyses</u>

The primary efficacy endpoints will compare the results from [^{68}Ga]FAPI-46 PET and histopathology. The table below displays the possible outcomes from the samples for each subject, with definitions for sensitivity and specificity.

		**Histopathology**	
		Positive	Negative
**[^{68}Ga]FAPI-46 PET**	Positive	A	B
	Negative	C	D
Total		A + C	B + D

Sensitivity = A / (A + C)

Specificity = D / (B + D)

The primary analysis of sensitivity and specificity will be summarized using frequency counts and percentages as well as 95% asymptotic normal confidence intervals. Inference for the primary hypotheses will be conducted utilizing a one-sample binomial proportion test with a normal approximation, a one-sided upper significance level of 0.05, and assumed null proportions/performance goals of 0.75 for sensitivity and 0.70 for specificity. Success for the primary efficacy analysis will be defined as demonstrating that the sensitivity is statistically significantly greater than the performance goal.

Accuracy of [^{68}Ga]FAPI-46 PET for the detection of FAP expressing cells compared to histopathology, defined as:

Accuracy = (A + D) / (A + B + C + D)

will be presented with a 95% confidence interval.

<u>Secondary Efficacy Analyses</u>

Association between SUV_{max} from [^{68}Ga]FAPI-46 PET on cross sectional imaging and FAP expression from IHC will be summarized by correlation coefficients with 95% confidence intervals.

1.2. Schema

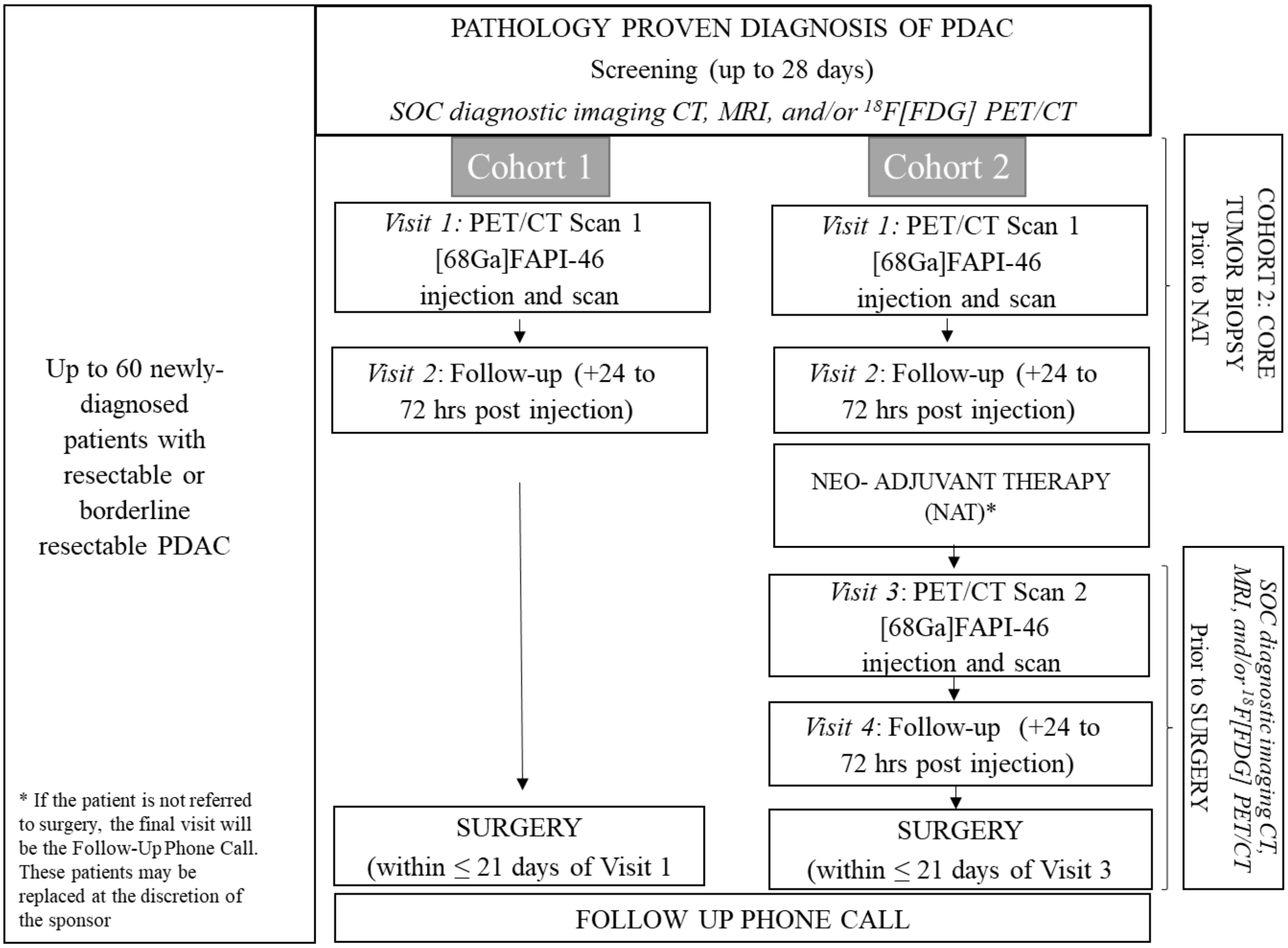
PATHOLOGY PROVEN DIAGNOSIS OF PDAC
Screening (up to 28 days)
SOC diagnostic imaging CT, MRI, and/or 18F[FDG] PET/CT
Up to 60 newly-diagnosed patients with resectable or borderline resectable PDAC
Cohort 1
Visit 1: PET/CT Scan 1 [68Ga]FAPI-46 injection and scan
Visit 2: Follow-up (+24 to 72 hrs post injection)
SURGERY (within ≤ 21 days of Visit 1
Cohort 2
Visit 1: PET/CT Scan 1 [68Ga]FAPI-46 injection and scan
Visit 2: Follow-up (+24 to 72 hrs post injection)
NEO- ADJUVANT THERAPY (NAT)*
Visit 3: PET/CT Scan 2 [68Ga]FAPI-46 injection and scan
Visit 4: Follow-up (+24 to 72 hrs post injection)
SURGERY (within ≤ 21 days of Visit 3
COHORT 2: CORE TUMOR BIOPSY Prior to NAT
SOC diagnostic imaging CT, MRI, and/or 18F[FDG] PET/CT Prior to SURGERY
FOLLOW UP PHONE CALL
* If the patient is not referred to surgery, the final visit will be the Follow-Up Phone Call. These patients may be replaced at the discretion of the sponsor

1.3. Schedule of Activities

Cohort 1: Participants who are considered surgical candidates by their doctor

Note: days are counted as calendar days unless otherwise specified. The day before study day 1 is Day -1.

Study Procedure	Screening	Visit 1	Visit 2[j]	Surgery	Surveillance Period (14 days)[h]
	≤ 28 days of D1	Study day 1	+ 24 to 72 hours post-injection	≤ 21 days of PET scan	
Informed consent	X				
Complete medical history, including surgical history	X				
Core biopsy diagnosis of PDAC	X				
Standard of care imaging: CT, MR and/or [^{18}F]-FDG PET	X				
Concomitant medications	X	X	X		
Physical examination [a]	X		X [f]		
ECOG performance status	X	X			
Vital signs [b]	X	X	X		
Serum chemistries [c]	X	X[k]	X		
CBC with differential		X[k]	X		
Tumor biomarkers (e.g., CA-19-9, CEA)[d]	X	As per standard of care			
Urine pregnancy test[e]	X	X[k]			
Baseline signs and symptoms		X			
Study drug [^{68}Ga]FAPI-46 administration and PET scan		X			
Assessment of tumor burden[d]	X				
Capturing administered treatments				X	X
AE assessment [i]		X	X		
Surgical resection and tissue collection				X[g]	

a: Includes height and weight. Height collected only during the screening window
b: Temperature, blood pressure, respiratory rate and pulse rate. Obtain twice on day of imaging: once prior to injection and once post-injection prior to discharge
c: Glucose, Na, K, Cl, Carbonate (CO_2), Mg, Ca, BUN, creatinine, AST, ALT, ALP, total protein, albumin, and total bilirubin
d: Frequency as per institutional standard of care; tumor burden assessment is obtained from surgical, pathology and/or imaging reports
e: As per institution's practice. If urine pregnancy test is inconclusive, a serum pregnancy test must be performed with results reviewed prior to study drug administration
f: Only if necessary for adverse event assessment; symptom directed exam is acceptable
g: IHC will be performed for FAP expression at a central lab on biopsy tissue; Histopathology for the detection of pancreatic cancer is performed locally
h: Passive follow-up only through chart review to collect information about post-surgical imaging, planned treatment, and outcomes for 14 days following surgery
i: AE assessments will be performed 24 hours post [^{68}Ga]FAPI-46 injection
j: Follow up visits may be performed through televisits. Patients may utilize institution approved local labs/mobile clinics for blood draws as part of follow up assessment. Subjects may enter surgery <24 hours post-injection if the time of Visit 2 follow-up assessment is 10 half-lives or more post [^{68}Ga]FAPI-46 injection
k. Labs obtained during screening period, within 72 hours of Day 1, can be used as pre-FAPI-46 injection Day1 lab data

Cohort 2: Participants who undergo NAT before being considered as surgical candidates by their doctor

Note: days are counted as calendar days unless otherwise specified. The day before study day 1 is Day -1.

**Study Procedure**	**Screen**	**Visit 1**	**Visit 2**[j]	**Neo-adjuvant Treatment Period**	**Visit 3**	**Visit 4**[j]	**Surgery**	**Surveillance Period (14 Days)**[h]
	≤ 28 days of D1	Study day 1	+ 24 – 72h post-injection		≤ 21 days of surgery	+ 24 – 72h post-injection		
Informed consent	X							
Complete medical history, including surgical history	X							
Core biopsy obtained prior to NAT		X						
Standard of care imaging: CT, MR and/or [^{18}F]-FDG PET	X				X[k]			
Record concomitant medications	X	X	X	X	X	X		
Physical examination [a]	X		X[f]		X[f]	X[f]		
ECOG Performance Status	X	X						
Vital signs [b]	X	X			X	X		
Clinical chemistry [c]	X	X[l]	X		X	X		
CBC with differential		X[l]	X		X	X		
Tumor biomarkers (e.g., CA-19-9, CEA)[d]	X							
Urine pregnancy test[e]	X	X[l]			X			
Baseline signs and symptoms		X			X			
Study drug [^{68}Ga]FAPI-46 administration and PET scan		X			X			
Assessment of tumor burden[d]					X			
Capturing administered treatments & procedures				X			X	X
AE assessments [i]		X	X		X	X[h]		
Surgical resection with tissue collection							X[g]	

a: Includes height and weight. Height measured only at screening.
b: Includes temperature, blood pressure, respiratory rate and pulse rate.
c: Assessments to include glucose, Na, K, Cl, Mg, Ca, BUN, creatinine, AST, ALT, ALP, total protein, albumin and total bilirubin.
d: Frequency as per institutional standard of care; tumor burden assessment is obtained from surgical, pathology and/or imaging reports
e: As per institution's practice. If urine pregnancy test is inconclusive, a serum pregnancy test must be performed with results reviewed prior to study drug administration
f: Only if necessary for adverse event assessment; symptom directed exam is acceptable
g: IHC will be performed for FAP expression at a central lab on biopsy tissue; Histopathology for the detection of pancreatic cancer is performed locally
h: Passive follow-up only through chart review to collect information about post-surgical imaging, planned treatment, and outcomes for 14 days following surgery
i: AE assessments will be performed 24 hours post [^{68}Ga]FAPI-46 injection
j: Follow up visits may be performed through televisits. Patients may utilize institution approved local labs/mobile clinics for blood draws as part of follow up assessment
k: Standard of care imaging post NAT Cycle 1 and prior to surgery. If multiple Standard of care images are taken post surgery, the one closest to the surgery date will be used for image analysis
l: Labs obtained during screening period, within 72 hours of Day 1, can be used as pre-FAPI-46 injection Day1 lab data

2. TABLE OF CONTENTS

Table of Contents

1. PROTOCOL SUMMARY 2
1.1. Synopsis 2
1.2. Schema 6
1.3. Schedule of Activities 7
Cohort 1: Participants who are considered surgical candidates by their doctor 7
Cohort 2: Participants who undergo NAT before being considered as surgical candidates by their doctor 8
2. TABLE OF CONTENTS 9
3. LIST OF ABBREVIATIONS AND DEFINITION OF TERMS 11
4. INTRODUCTION 12
4.1. Background 12
4.2. Unmet Need 12
4.3. Investigational Product: [^{68}Ga]FAPI-46 13
4.4. Nonclinical and Previous Clinical Studies Overview 14
4.5. Rationale for this Study 15
5. STUDY OBJECTIVES AND ENDPOINTS 15
5.1. Objectives 15
5.2. Endpoints 16
6. STUDY DESIGN AND SCHEMATIC 16
6.1. Study Design 16
6.2. Patient Population 18
6.3. Study Intervention 19
6.4. Study Intervention Discontinuation and Participant Discontinuation/withdrawal 20
7. STUDY ASSESSMENTS AND PROCEDURES 22
7.1. Study Procedures 22
7.2. Adverse Events 25
7.3. Unanticipated Problems 28
8. STATISTICAL ANALYSIS 30
8.1. General 30
8.2. Safety Analysis 30
8.3. Efficacy Analysis 31
8.4. Pharmacodynamic Analysis 32
8.5. Tumor Response 32

8.6. Interim Analysis 32

8.7. Sample Size 32

9. SUPPORTING DOCUMENTATION AND OPERATIONAL CONSIDERATIONS 32

9.1. Regulatory, Ethical, And Study Oversight Considerations 32

9.2. Source Documentation 38

9.3. INVESTIGATOR AGREEMENT 41

10. REFERENCES 42

11. APPENDICES 43

11.1. Appendix 1: Serious Adverse Event Reporting 43

11.2. Appendix 2: Eastern Cooperative Group (ECOG) Performance Status Scale 44

11.3. Appendix 3: Response Evaluation Criteria in Solid Tumors (RECIST 1.1) QUICK REFERENCE 45

11.4. Appendix 4 National Cancer Institute Common Terminology Criteria for Adverse Events (NCI CTCAE) Version 5.0 50

3. LIST OF ABBREVIATIONS AND DEFINITION OF TERMS

**AE**	Adverse Event
**CFR**	Code of Federal Regulations
**eCRF**	Electronic Case Report Form
**CRO**	Contract Research Organization
**CBC**	Complete Blood Count
**CTCAE**	Common Terminology Criteria for Adverse Events
**EC**	Ethics Committee
**EDC**	Electronic data capture, synonymous with the study database
**FAPI**	Fibroblast-activation-protein inhibitors
**FDA**	U.S. Food and Drug Administration
**GCP**	Good Clinical Practice
**IB**	Investigator's Brochure
**ICF**	Informed Consent Form
**ICH**	International Council for Harmonisation of Technical Requirements for Pharmaceuticals for Human Use
**IHC**	Immunohistochemistry
**IRB**	Institutional Review Board
**MedDRA**	Medical Dictionary for Regulatory Activities
**NAT**	Neoadjuvant therapy (chemotherapy, radiation therapy, or both)
**NPV**	Negative Predictive Value
**PET**	Positron Emission Tomography
**PPV**	Positive Predictive Value
**SAE**	Serious Adverse Event
**SUSAR**	Suspected Unexpected Serious Adverse Reaction
**SUV**	Standardized Uptake Value

4. INTRODUCTION

4.1. Background

Pancreatic ductal adenocarcinoma (PDAC) is an aggressive disease with high morbidity and mortality rates coupled with significant patient management challenges due to diagnostic options that may miss established or developing metastatic disease, or lead to interpretive errors. PDAC management is further complicated by limited options to evaluate therapeutic approaches in an interval that allows appropriate patient management and intervention. PDAC aggressive nature is highlighted by 2021 estimates where 60,430 new cases of pancreatic cancer are expected to be diagnosed in the US with 48,220 deaths from the disease; as expected, worldwide cases significantly amplify these numbers confirmed by 2020 statistics that revealed 495,773 new cases with 466,003 deaths. At present, there is no screening or early detection test for pancreatic cancer that has been shown to reduce mortality from the disease. Surgery, radiation therapy, and chemotherapy remain treatment options and may extend survival and/or relieve symptoms, but seldom are curative; importantly, because of the absence of reliable tests for early detection, fewer than 20% of patients are candidates for surgery since the cancer has already extended beyond the pancreas when it is finally and accurately diagnosed. Adjuvant treatment may lower the risk of recurrence for those who have surgery, however for all stages combined, the 5-year relative survival rate remains very dismal at ~10% [American Cancer Society, 2021].

Due to its low to absent expression in healthy tissues, increased expression in focal wound healing, inflammation, and fibrosis, and very high expression in Cancer-Associated Fibroblasts (CAFs) found in tumor stroma (a component of the tumor microenvironment) and in specific tumor tissues, impressive preliminary published data for FAP and FAP inhibitors (FAPI) have become an important and very promising diagnostic target in cancer. Besides the clear diagnostic potential because of FAP expression on CAF and direct expression on tumor cells, FAP may be an attractive therapeutic target, alone or in combination with other therapies that directly target the tumor, including chemo-, immunologic, radiation-, or cell-based therapies whose function may be otherwise blunted or blocked by the tumor stroma. An effective marker of FAP expressing cells would provide significant value in identifying patients who might benefit from FAP therapeutic applications in addition to potentially monitoring impact to other, non-FAP, therapies.

We are proposing clinical studies to investigate [^{68}Ga]FAPI-46 PET imaging's diagnostic performance, accuracy, sensitivity and specificity in detecting FAP expressing cells in patients with PDAC.

4.2. Unmet Need

Rapid advancements in imaging technologies, particularly PET, have been made in recent decades and PET imaging is now a fundamental part of patient management in oncology practice. Although [^{18}F]Fluorodeoxyglucose (FDG) is the most commonly used PET agent for the diagnosis, staging and assessment of treatment responses in many solid tumors, the use in pancreatic cancer has not been universally adopted. According to the published literature, the clinical experience with FDG PET in staging pancreatic cancer has a 64% sensitivity according to a meta-analysis of 4 studies with a 95% confidence interval ranging from 0.50 to 0.76 [Wang et al, 2013]. Thus, in clinical staging of pancreatic cancer, FDG is not ideal.

Given these limitations of current staging methods in pancreatic cancer patients that are resectable or borderline resectable, additional strategies have been sought to fulfill this continued need for more sensitive imaging agents to detect occult malignant disease.

4.3. Investigational Product: [^{68}Ga]FAPI-46

4.3.1. Development and Optimization

While numerous FAPI (fibroblast activation protein inhibitor) compounds have been developed in recent years, the rational design, development and improvement of these molecules were focused on achieving high tumor retention, high signal-to-background, high tumor uptake, and low activity in normal organs. This iterative work was very successful and resulted in FAPI-46 being identified as the preferred compound for radiolabeling to best meet the criteria and requirements noted above--allowing optimal compound selection for further development.

Initial work by Loktev et al. 2019 on a PET imaging radiotracer targeting FAP (fibroblast activation protein) produced the FAPI-01 and FAPI-02 compounds. From this work, FAPI- 02 showed superior performance with higher cellular uptake and retention time. Further clinical work evaluating [^{68}Ga]FAPI-02 in three patients further confirmed specificity in tumors with high image contrast.

Subsequent work by Lindner et al. 2019 focused on necessary criterium for prolonging tumor retention time, resulting in the development of fifteen new FAPI compounds; the best of which for imaging and diagnosis was FAPI-04. Through a series of experiments utilizing FAP-expressing cells and small animal imaging, these FAPI compounds were evaluated based on cell uptake, retention, specificity to FAP versus a serine protease from the same family (DPP4), biodistribution, and tumor-to-organ ratios. Results indicated that FAPI-04 was, at that time, the better candidate showing rapid internalization into FAP-expressing tumors and favorable tumor-to-organ ratios. Moreover, FAPI-04 was a marked improvement over FAPI-02 with a significantly improved tumor retention over 24 hours. Kratochwil et al. 2019 further demonstrated the utility of FAPI-04 in a groundbreaking study in 80 patients, with 28 different types of cancer, that were successfully imaged with [^{68}Ga]FAPI-04, showing high image contrast and good tumor delineation.

4.3.2. Selection of FAPI-46 for development

Because of these improved and continued promising results with FAPI-04, further optimization to enhance tumor retention while maintaining high image contrast was identified, needed and feasible. As an extension to their early work noted above, Loktev et al. 2019 developed and tested fifteen additional FAPI compounds which identified FAPI-46 as having marked improved performance, including better tumor uptake and higher tumor-to-organ ratios compared to FAPI-04, confirming FAPI-46 as the preferred, optimal compound for FAP PET imaging.

In addition to the work aimed at optimizing FAPI-compounds, several non-clinical *in vitro* and *in vivo* studies have been conducted to specifically investigate FAPI-compounds in pancreatic cancer. Loktev et al. 2019 and Watabe et al. 2020 demonstrated negligible binding of FAPI to the human pancreatic ductal adenocarcinoma (PDAC) cell lines BxPC3, Capan-2, PANC-1 and MIA PaCa *in vitro*. Conversely, in mice bearing subcutaneous Capan-2, PANC-1 or MIA PaCa tumors, [^{68}Ga]FAPI tumor uptake was confirmed and may be explained by the recruitment and subsequent activation of mouse fibroblasts. As expected, immunohistochemistry showed FAP expression in the stroma of these xenografts. Lastly, a PDAC patient-derived xenograft and orthotopically implanted KP4662 PDAC tumors were imaged using [^{68}Ga]FAPI-46 and exhibited tumor uptake matched by FAP positivity in the stroma per immunohistochemistry (unpublished data, University of California Los Angeles [UCLA]).

4.3.3. Investigational Product Overview

The investigational product is [^{68}Ga]FAPI-46.

4.3.4. Structure:

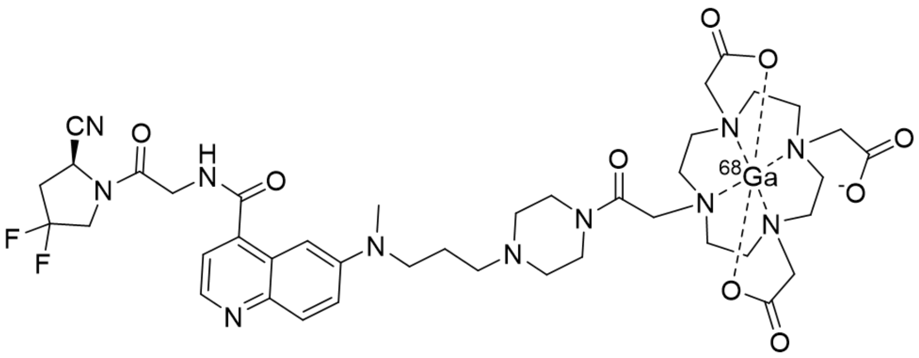

4.3.5. Synthesis

FAPI-46 precursor is a small molecule critical drug intermediate consisting of a FAPI moiety conjugated with a DOTA chelator for use in manufacturing the radiolabeled final drug product. FAPI-46 precursor is supplied in single use vials (0.050 mg ± 20%) from ABX in powder form. The radionuclide in this study, gallium-68, is a neutron deficient isotope (31 neutrons and 37 protons) of gallium which decays via electron capture and positron emission to zinc-68. Gallium-68 has a 68-min. half-life, with a relatively low translational energy of the positron (1.9 MeV) for producing high quality positron emission tomography (PET) images. FAPI-46 precursor is reacted with gallium-68 to produce the final drug product [^{68}Ga]FAPI-46. The resulting material contains buffering excipients that maintain the stability to produce the [^{68}Ga]FAPI-46 final drug product. [^{68}Ga]FAPI-46 final drug product will be manufactured and tested by Sofie Network Inc.

4.4. Nonclinical and Previous Clinical Studies Overview

See Investigational Brochure for additional information.

Preclinical studies by Loktev et al. 2019 established that FAPI-46 as an extremely promising PET tracer with high binding specificity and selectivity to FAP-expressing cells, with high tumor-to-healthy tissue contrast. Radiolabeled FAPI-46 serum stability study showed that at 24 hours only the intact radioactive drug was detected with neither radioactive degradation products nor free radioactivity being observed or measured, supporting *in vivo* stability in human sera. In cell culture experiments, FAPI-46 was incubated with cells to assess binding affinity and specificity. FAPI-46 was shown to exhibit strong binding to HT-1080-hFAP cells with an EC_{50} of 13.5 nM. Negligible binding was noted in a cell line not expressing FAP.

In vivo pharmacodynamic studies were conducted to demonstrate *in vivo* and *ex vivo* biodistribution, as well as *in vivo* binding specificity. *In vivo* and *ex vivo* biodistribution data were highly congruent with a high tumor:blood, tumor:liver and tumor:muscle ratios. Binding specificity was confirmed *in vivo,* whereby the addition of unlabeled compound competitively blocked all [^{68}Ga]FAPI-46 accumulation in tumors.

A single dose, acute and extended, Good Laboratory Practice (GLP) compliant toxicology study was performed. A single dose of [^{nat}Ga]FAPI-46 (166.7 µg/kg) was administered intravenously to male and

female rats. Acute and delayed toxicity evaluations were performed. The dose was well-tolerated and no adverse effects were noted. The human equivalent dose (HED) was preselected as 100 µg for a 60 kg adult which is 100 x of the no observed adverse effect level (NOAEL) established in rats.

Data regarding the safety, dosimetry and efficacy of [^{68}Ga]FAPI-46 in humans have been extracted from published literature and has been received as personal communications from cited investigators and institutions. [^{68}Ga]FAPI-46 has been evaluated for biodistribution and dosimetry [Meyer et al, 2019]. [^{68}Ga]FAPI PET/CT imaging was shown to be safe for administration up to at least 200 MBq (5.4 mCi) [Meyer et al., 2019]. At 200 MBq (5.4 mCi), the whole-body effective dose including CT is 5.3 mSv, with CT contributing 3.7 mSv. Next, biodistribution was assessed for two of the six patients (one male and one female) [Meyer et al, 2019]. The highest average normal organ SUVmax at all time points was for the liver.

Tracer uptake in the tumor was rapid and had a greater retention time than normal organs; however, both mean SUVs decreased in all patients from the first to the last time points and tumor-to-background ratios increased over time. Therefore, in the totality of all the data, it was clearly determined that [^{68}Ga]FAPI-46 has a favorable dosimetry, avidity, and diagnostic isotope labeling profile with a biodistribution profile amenable for detection of FAP- expressing cells in patients with PDAC.

Lastly, but importantly, from preliminary diagnostic patient studies to date, there have been > 200 [^{68}Ga]FAPI-46 PET/CT scans with no reported adverse events [K Hermann, Personal Communication. Essen University Hospital].

4.5. Rationale for this Study

As a diagnostic radiopharmaceutical at minute tracer amounts, the very favorable dosimetry and non-toxic molecular design, a very robust benefit/risk and safety profile is expected and will be studied and confirmed during further product development.

5. STUDY OBJECTIVES AND ENDPOINTS

5.1. Objectives

5.1.1. Primary Objective

- Evaluate the performance [sensitivity, specificity, accuracy] of [^{68}Ga]FAPI-46 PET imaging to detect FAP-expressing cells, using histopathology as truth standard.

5.1.2. Secondary Objectives

- Evaluate positive and negative predictive values of [^{68}Ga]FAPI-46 PET images, to detect FAP-expressing cells, using histopathology as truth standard.
- Correlate the histopathology with FAP staining on FAP IHC assay.
- Further characterize the safety profile of [^{68}Ga]FAPI-46 in patients with PDAC

5.1.3. Exploratory Objectives

- Compare the detection of local and metastatic disease using [^{68}Ga]FAPI-46 PET to a composite of clinical, radiological (i.e. CT, MR), and histopathological reference in

patients with resectable or borderline resectable PDAC.

- Compare pre- and post-treatment [^{68}Ga]FAPI-46 PET evaluations obtained in Cohort 2 to identify perturbations, if any, from neoadjuvant therapy treatment.

5.2. Endpoints

5.2.1. Primary Efficacy Endpoint

- Sensitivity, specificity, and accuracy will be determined on a per-lesion basis for all lesions with tissue available for analysis.
    - Lesions positive for FAP as detected by [^{68}Ga]FAPI-46 PET imaging
    - Malignant lesions as detected by histopathology

5.2.2. Secondary Efficacy Endpoints

- Positive and negative predictive value will be determined on a per-lesion basis for all lesions with tissue available for analysis.
    - Lesions positive for FAP as detected by [^{68}Ga]FAPI-46 PET imaging
    - Malignant lesions as detected by histopathology
- Staining intensity assessed by immunohistochemistry, using H-score, correlated to the uptake of [^{68}Ga]FAPI-46, using SUV_{max}.
    - Lesions positive for FAP as detected by immunohistochemistry

5.2.3. Exploratory Efficacy Endpoints

- Number of malignant lesions (local, metastatic) identified through anatomical (i.e, CT, MR) and/or [1^{8}F]-FDG PET
- Number of [^{68}Ga]FAPI-46 identified lesions and the radiotracer accumulation observed in local and metastatic disease using SUVPercentage change in [^{68}Ga]FAPI-46 PET imaging using SUV levels and number of identified lesions between pre- and post-neoadjuvant therapy.

5.2.4. Safety endpoints

- Incidence and severity of TEAEs or TESAEs 24 hours from [^{68}Ga]FAPI-46 administration according to MedDRA/CTCAE V.5.0.

6. STUDY DESIGN AND SCHEMATIC

Protocol waivers or exemptions are not allowed with the exception of immediate safety concerns. Therefore, adherence to the study design requirements, including those specified in the outline of study procedures are essential and required for study conduct.

6.1. Study Design

This is a prospective, multi-center, single arm, open label, non-randomized study to evaluate the ability of [^{68}Ga]FAPI-46 to detect FAP expressing cells in patients with resectable or borderline resectable PDAC. The [^{68}Ga]FAPI-46 PET scans will be acquired after initial staging using institutional standard methods. If the participant is prescribed neoadjuvant therapy, a second [^{68}Ga]FAPI-46 PET scan will be performed within 21 days prior to planned surgical resection. This will be followed by histopathology and IHC analyses and comparison to resected PDAC tumor specimens. The surgeon will not be blinded to the [^{68}Ga]FAPI-46 scans prior to surgery.

Experimental Design: Phase 2, prospective, multi-center, single arm, non-randomized, open label study
Allocation of treatment: open-label, non-randomized, single intervention

Number of patients planned: Up to 60 patients will be enrolled. Participants who withdraw, are withdrawn, do not complete the [^{68}Ga]FAPI-46 PET scan ≤ 21 days prior to surgery (Cohort 2), do not have a core biopsy prior to NAT or who are not surgical candidates post-NAT (Cohort 2) may be replaced at the discretion of the sponsor.

Summary of Study Visits and Data Collection: Refer to Schedule of Events for full requirements for each visit. The Schema provides a graphical representation of the study visits.

All participants

Screening: Screening assessments can be conducted at multiple visits provided they are within 28 days prior to study day 1, including anatomic imaging. Tests performed for standard of care may be used for screening purposes provided they are within window.

Visit 1: Defines Study Day 1. [^{68}Ga]FAPI-46 PET scan obtained.

Visit 2: 24 to 72 hours after [^{68}Ga]FAPI-46 injection. Safety assessment with safety labs and, if needed, a symptom directed physical examination . This is the last active study-related event with the participant.

Cohort 1 only: Participants in Cohort 1 are those not prescribed neoadjuvant therapy and directly undergo surgical resection within 21 days post-[^{68}Ga]FAPI-46 PET scan. Data will be mined from the medical records regarding the surgical procedure and pathologic outcomes. The final study-directed visit is Visit 2.

Surgery: Surgical resection to occur within 21 days after [^{68}Ga]FAPI-46 PET scan. Resected tissue should be collected as per laboratory manual. Surgical and pathology reports will be collected, codified, and provided for study.

Follow-up: Passive follow-up only through chart review to collect information about post-surgical imaging, planned treatment, and outcomes for 14 days following surgery.

Cohort 2 only: Participants in Cohort 2 are those who receive a tumor biopsy, are prescribed neoadjuvant therapy followed by surgical resection within 21 days after a second [^{68}Ga]FAPI-46 PET scan. Neoadjuvant therapy is beyond the scope of this protocol and is to be administered per standard of care by the institution and treating oncology team. Data will be mined from the medical records regarding the neoadjuvant therapy administered, surgical procedure and pathologic outcomes. The final study-directed visit is Visit 4.

Visit 3: Second [^{68}Ga]FAPI-46 PET scan.

Visit 4: 24 to 72 hours after [^{68}Ga]FAPI-46 injection. Safety assessment with safety labs and, if needed, a symptom directed physical examination.

Surgery: Surgical resection to occur within 21 days after [^{68}Ga]FAPI-46 PET scan. Resected tissue should be collected as per laboratory manual. Surgical and pathology reports will be collected.

Follow-up: Passive follow-up only through chart review to collect information about post-surgical imaging, planned treatment, and outcomes for 14 days following surgery.

6.2. Patient Population

6.2.1. Sample Size

Up to 60 patients with resectable or borderline-resectable pancreatic ductal adenocarcinoma (PDAC) cancer who may be considered candidates for surgical resection without or with neoadjuvant treatment will be enrolled in the study.

6.2.2. Inclusion Criteria

Patients must meet **ALL** criteria listed below for entry:

1. Patients with pathologically confirmed pancreatic ductal adenocarcinoma.
2. Treatment-naïve
3. Staged as resectable or borderline-resectable
4. Planned to undergo surgical resection or to receive neoadjuvant therapy (i.e., chemotherapy, radiation therapy, or combination) and subsequent planned surgical resection
5. Anatomic imaging (e.g., CT, MRI) obtained within ≤ 28 days of consent
6. Age ≥ 18 years
7. Completed informed consent as determined per the IRB of record

6.2.3. Exclusion Criteria

Patients will be excluded from entry if **ANY** of the criteria listed below are met:

1. Pregnant as determined by a pregnancy test as per institutional guidelines for individuals of child-bearing potential
2. Declining to use effective contraceptive methods during the study (for individuals of child-producing potential)
3. Need for emergent surgery that would be delayed by participation
4. Bacterial, viral, or fungal infections requiring systemic therapy, that are expected to impact FAP expression in the opinion of the sponsor or their designee
5. Serious co-morbidities and serious nonmalignant disease (e.g., hydronephrosis, kidney failure, liver failure, systemic or local inflammatory or autoimmune diseases or other conditions) that in the opinion of the investigator, physician of record and/or Sofie could compromise patient safety and/or protocol objectives
6. Known diagnosis of an autoimmune disorder, that is expected to impact FAP expression in the opinion of the sponsor or their designee
7. Patients receiving any other investigational agent within the past 28 days
8. Breastfeeding. Note: nursing parents are allowed if the potential participant commits to pumping breast milk and discarding it from injection to ≥ 24 hours from the time of the [^{68}Ga]FAPI-46 injection.
9. Known hypersensitivity to any excipients used in [^{68}Ga]FAPI-46: trace amounts of sodium acetate sodium ascorbate and/or hydrochloric acid

6.2.4. Concomitant Medication

No other investigational medical products are allowed during this study, from eligibility determination to surgery. Participant may receive investigational medical products post-surgery for the purposes of another clinical trial.

Drugs used off-label consistent with institutional practice, standard of care, or practice of medicine do not meet the definition of investigational medical product.

Participants should receive full concomitant care during this clinical trial consistent with institutional practices and standard of care.

6.3. Study Intervention

6.3.1. [^{68}Ga]FAPI-46

FAPI-46 precursor is reacted with gallium-68 to produce the final drug product [^{68}Ga]FAPI-46. The resulting material contains buffering excipients that maintain the stability to produce the [^{68}Ga]FAPI-46 final drug product.

6.3.2. Dosing and Administration

Intravenous administration of radiolabeled [^{68}Ga]FAPI-46 (5 mCi ± 2 mCi) as per institutional policies, administered 15 minutes (±10 minutes) prior to the initiation of the attenuation correction CT.

6.3.3. Preparation, Handling, Storage, and Accountability

- **Acquisition and accountability**

An accurate and current accounting of the precursor and/or study drugs will be maintained utilizing an ALCOA compliance drug accountability record (DAR) that will be verified by the Sponsor's study monitor.

Drug accountability will be documented using the study's drug accountability and receipt form (DARF) or the investigational site's template *if* deemed acceptable by the sponsor and/or designee. Documentation confirming review and approval of the Site's template must be provided by Sponsor or designee and filed within the ISF.

At minimum:

- Receipt: date received and signature/mark individual confirming receipt
- Dispensing: date, quantity of IMP (both in ml and mCi), study identification number and initials of participant, and signature/mark of individual confirming these dispensing details.
- Investigational medical product destroyed, where 'destroyed,' is equivalent to allowing decay for 10 half-lives and then destroying the physical component consistent with institutional policy & procedure.

The DAR will be monitored against study drug and supply shipments and ALCOA.

Drug components provided by the Sponsor or designee must be used only for this study. Accountability logs must be utilized to track drug components.

Only authorized prescribers may prescribe the investigational medical product ^{68}Ga-FAPI-46.

- **Authorized prescribers must be authorized users as per NRC.**

A copy of the prescription must be kept on file in the participant's medical record or, if precluded by institutional policy, within the study binder.

- **Formulation, appearance, packaging and labeling**

The final [^{68}Ga]FAPI-46 drug product (DP) is a clear, colorless, and particulate free, sterile solution supplied in 10 mL Type 1 glass vial. The DP contains 0.1 mCi/ml to 5.0 mCi/ml [^{68}Ga]FAPI-46 at the end of synthesis (EOS) and 11.81 mg/mL Sodium Acetate and 0.49 mg/mL Sodium Ascorbate in sterile water for injection in approximately 9.9 ml.

- **Storage and stability**

Stored upright in lead-shielded container at 15 – 25 °C with use before expiration of 4 hours post end of synthesis (EOS). Handling of the investigational medical product is subject to radioactive material regulations and licensing requirements.

- **Preparation**

[^{68}Ga]FAPI-46 is provided as ready to use vial or syringe. No further preparation or manipulation is required. The dose will be administered to the patient according to the site's standard operating practices.

6.3.4. Intervention Compliance

All interventions in this protocol are administered by licensed medical staff under the supervision of the study investigator. For this reason, adherence to protocol will be assessed through source document verification in the electronic health records for:

- Prescription, dispense, and administration of [^{68}Ga]FAPI-46
- Scan settings (e.g. bed positions, field of view settings)

6.4. Study Intervention Discontinuation and Participant Discontinuation/withdrawal

6.4.1. Discontinuation of Study Interventions

Participants who withdraw after consenting to the clinical trial, but do not receive an injection of [^{68}Ga]FAPI-46, will not be followed for this clinical trial.

Participants who withdraw after receiving at least 1 injection of [^{68}Ga]FAPI-46 but who are not scanned will be followed as an intent-to-treat population for the purposes of safety assessments. If the participant withdraws full permission (i.e., revokes HIPAA authorization), the participant will not be followed further, but all data obtained until the date of HIPAA revocation will be used for the purposes of this study. Participant's intent must be well documented in the case book.

A participant's discontinuation of the study intervention (i.e., declining the second [^{68}Ga]FAPI-46 scan for Cohort 2) does not immediately define the subject as being off-study. **The Sponsor should be contacted to determine if study procedures should continue should the participant consent to it.** Participants who decline further intervention (i.e. scans) should be evaluated by the Sponsor as an unanticipated problem and/or serious adverse event.

Scenarios for study intervention discontinuation:

- **Participant declines further study intervention**. If a participant who has received at least one injection of [^{68}Ga]FAPI-46 declines further study intervention, the reason for declining further participation should be documented – specifically, if it is due to an adverse event.
- **Unforeseen medical event.** If a participant undergoes a medical event that compromises the subject's safety or study's endpoints, the participant may be withdrawn from treatment. The sponsor or investigator may make this determination. If a participant is withdrawn for this reason, the

subject must be followed for a minimum of 24 hours from the last injection with investigational product.

- **Pregnancy**. If a participant declares pregnancy, the participant will be withdrawn from study procedures. The Sponsor will be notified within 1 business day of the event. The participant should be asked to consent to have the pregnancy followed (as appropriate).
- **Participant non-compliance**. If a participant does not comply with the required adverse event assessment (i.e., hematologic and serum chemistry assessments, follow-up assessments), study treatment may be discontinued to reduce risk to the participant.

6.4.2. Participant Discontinuation / Withdrawal from the Study

Subject participation in the study may be discontinued entirely (i.e. withdrawn from study without further follow-up or interaction) in the following circumstances:

- **Participants who decline to receive any study intervention after consenting** to the clinical trial are withdrawn from study and are not followed for this clinical trial.
- **Investigator non-compliance**. If an investigator is considered to be non-compliant with the study protocol or federal, state, local, or institutional requirements, the sponsor may end the study and withdraw subjects from trial.
- **Participant's refusal to continue**. Participants who decline further study follow-up (i.e. no longer wish to undergo study assessments) are withdrawn from the study. If the participant withdraws full permission (i.e., revokes HIPAA authorization), the participant will not be followed further, but all data obtained until the date of HIPAA revocation will be used for the purposes of this study. Subject's intent must be well documented in the case book.
- **Termination of study**. If the study is terminated for any reason, the participant will be followed for a minimum of 24 hours from the last injection of [^{68}Ga]FAPI-46 for safety assessment, including hematologic and serum chemistries as prescribed per protocol.

The reason for participant discontinuation or withdrawal from the study will be recorded in the subject's casebook and entered into the electronic data capture system (EDC).

6.4.3. Lost to Follow-Up

The following actions must be taken if a participant fails to return to the clinic for a required study visit:

- **Imaging Visit**. If the participant fails to maintain the scheduled appointment for the ^{68}Ga-FAPI-46, and the investigational medical product is wasted, the site will contact the Sponsor or designee. The Sponsor or designee will determine if the participant should be removed from study or be allowed the opportunity to be rescheduled.
- **Safety follow-up**. The investigator or designee will make every effort to regain contact with the participant (where possible, 3 telephone calls and, if necessary, a certified letter to the participant's last known mailing address or local equivalent methods). These contact attempts should be documented in the participant's medical record or study file.

Should the participant continue to be unreachable, he or she will be considered to have withdrawn from the study with a primary reason of lost to follow-up.

6.4.4. Replacement Criteria

Participants who have withdrawn or are withdrawn from study may be replaced at Sponsor's discretion.

7. STUDY ASSESSMENTS AND PROCEDURES

An overview of the study schedule is provided in tabular form under "SCHEDULE OF EVENTS." The timing of each visit will be relative to prior visit. Individual study procedures are described below in Section 7.1.

7.1. Study Procedures

7.1.1. Informed Consent

Informed consent will be obtained prior to undertaking any protocol-specific research-only procedures. All participants must provide informed consent as outlined by the IRB of record. No elements of consent will be waived and will be compliant with U.S. FDA federal code.

If written consent is used, the participant must receive a copy of the signed informed consent form. If oral consent is used, the participant must be provided documentation as outlined by the IRB of record. Documentation of the consent process must be maintained in the participant's medical record or the participant's case history, dependent upon institutional policies.

7.1.2. Contraception

For individuals of child-bearing potential, effective contraception must be utilized during the study. Effective contraceptives include abstinence as well as barrier and oral contraceptives.

If participants are engaging in fertility sparing strategies (i.e. ova collection and banking, sperm collection and banking), this should be done prior to injection of study agent.

Participants should not discontinue contraceptive strategies without speaking with their treating oncologists.

7.1.3. Medical and Surgical History

The following should be documented in the participant's case history and reported through the EDC:

- Medical history related to fibroinflammatory diseases, cancers, or any conditions which may impact uptake of the study drug, as documented in the medical record for the 5 years (e.g. such as non- rheumatoid arthritis, gout, edema). Medical history should provide explanation / justification for any active treatments and concomitant therapies taken at study entry.
- Known allergies
- History of myocardial infarction or other life-threatening event, regardless of time-frame.
- Race and ethnicity; if participant declines, this should be clearly noted in the case history.
- Any prior radiation and its indication.

7.1.4. Concomitant Medications

For the purposes of this protocol, a concomitant therapy is considered to be any of the following:

- Prescription medication from an authorized prescriber
- Over the counter medications
- Complementary or alternative medications, including herbal remedies or supplements

A concomitant medication log, separate from the participant's medical chart, will be maintained for the purposes of this trial. This log will be a source document and must meet ALCOA standards. Appropriate medical staff will review concomitant medications at each study visit with the subject so the medications can be reviewed if an untoward event occurs during the study.

All concomitant medications and supportive therapy administered from consent (i.e. routine medications) through end of must be reported using on the appropriate case report form (eCRF).

7.1.5. Routine and Symptom Directed Physical Examination

A physical examination is required per protocol for study eligibility during the screening window. At minimum, the physical examination includes examination of major body systems, including general appearance, skin, neck, head, eyes, ears, nose, throat, heart, lungs, abdomen, lymph nodes, extremities and nervous system.

A symptom focused physical exam is performed as needed during the adverse event window 24 to 72 hours post-injection of [^{68}Ga]FAPI-46. This physical exam would be performed to further characterize an observed adverse event for study documentation. The adverse event or concern triggering symptom-focused physical exam should be clearly documented.

7.1.6. Vital Signs

For protocol, collected vital signs are blood pressure, heart rate, respiratory rate, temperature, and weight. Vital signs must be obtained after sitting at least two minutes; time seated and time obtained must be documented. Height is collected at screening only.

On day of [^{68}Ga]FAPI-46, a set of VS must be obtained prior to injection and post-injection prior to discharge.

7.1.7. Performance Status

Performance status will be assessed using ECOG performance status criteria as outlined in Appendix 2.

7.1.8. Clinical Laboratory Tests

Local laboratories will analyze all hematology, blood chemistry collected for the study. Samples will be analyzed at a facility meeting Clinical Laboratory Improvement Amendments (CLIA) requirements and/or using methods documented in a methods validation report.

7.1.9. CBC with differential

White blood cell count, red blood cell count, hematocrit, hemoglobin, platelets, absolute neutrophil count, lymphocytes, basophils, eosinophils, and monocytes will be collected at screening and per the schedule of events.

CBC w/diff must be resulted prior to injection with [^{68}Ga]FAPI-46.

7.1.10. Serum Chemistry

Glucose (non-fasting), sodium, potassium, chloride, carbon dioxide, magnesium, calcium, blood-

urea-nitrogen (BUN), creatinine, aspartate aminotransferase, alanine aminotransferase, alkaline phosphatase, total protein, albumin, and total bilirubin.

7.1.11. Pregnancy Test(s)

A urine pregnancy test will be performed for individuals of child-bearing potential at screening and prior to injection of [^{68}Ga]FAPI-46 as per institutional policies, but no more than 72 hours of dosing study drug.

If the urine pregnancy test is inconclusive, a blood test must be ordered and resulted as negative prior to injection of [^{68}Ga]FAPI-46.

An individual who has recently undergone an abortive procedure may still have elevated hCG levels. In this situation, documentation from a treating physicians that the patient is not pregnant will suffice *in lieu* of a pregnancy test.

7.1.12. [^{68}Ga]FAPI-46 PET Scan

The imaging manual contains detailed technical information for the [^{68}Ga]FAPI-46 PET/CT Scan.

7.1.13. Prior Preparation

Participants should be directed to hydrate well the day prior to scan. The scan does not require fasting but participants are asked to abstain from alcohol and supplements ~ 1 day prior to scan. Participants should rest well the night before the scan.

7.1.14. Day of Scan

Obtain vitals (section 7.1.5) prior to initiating procedures to reduce stress-associated elevations.

[^{68}Ga]FAPI-46 is administered intravenously; intravenous access is per institutional standard of care for administration of PET tracer agents.

During the uptake period (15 minutes (±10 minutes), participants should be encouraged to hydrate and void frequently. Participants should be directed to void prior to being placed on the scanner.

Scanning will be initiated 15 minutes (±10 minutes) post-injection. Scanner coverage should be set up to extend from vertex to mid-thighs. Low dose CT is to be used for anatomical correlation.

For Cohort 2, the same scanner must be used for the two scans unless there is a scanner failure or other technical issue.

7.1.15. Tissue Specimen Collection and Handling

Tissues collected at biopsy or surgery will be processed according to institutional standard for local histopathological assessment of pancreatic cancer.

See Laboratory Manual for detailed instructions for the preparation, packaging, and shipping instructions for tumor tissue that will be evaluated by IHC for FAP evaluation.

7.1.16. Tumor Burden Assessment

Disease burden will be assessed using RECIST criteria by a central imaging core lab. Local imaging (e.g. CT, MRI, PET/CT) will be anonymized eliminating the PHI, codified with participant study ID, and will be provided to the Sponsor/CRO for third party imaging analysis. Imaging to be submitted is outlined in the Schedule of Activities.

7.2. Adverse Events

7.2.1. Collection Window

For the purposes of this study, the adverse event collection window is from the time of injection with [^{68}Ga]FAPI-46 through 24 hours post-injection. If the 24 hour window ends on a holiday or non-clinic day (or the participant cannot return to clinic due to unforeseen circumstances, such as weather), the window may be extended until 72 hours post-injection.

Adverse event collection windows are tied to the number of [^{68}Ga]FAPI-46 injections. Cohort 1 will have one AE collection window whereas Cohort 2 will have two discrete AE collection windows.

Any adverse event suspected to be related to [^{68}Ga]GAPI-46 – regardless of collection window – should be assessed and reported to the Sponsor/CRO

7.2.2. Definitions

This study defines terms as per the U.S. federal code at 21CFR§312.32:

- **Adverse event**

Any untoward medical occurrence associated with the use of a drug in humans, whether or not considered drug related.

- **Life-threatening adverse event or life-threatening suspected adverse reaction**

An adverse event or suspected adverse reaction is considered "life-threatening" if, in the view of either the investigator or sponsor, its occurrence places the patient or subject at immediate risk of death. It does not include an adverse event or suspected adverse reaction that, had it occurred in a more severe form, might have caused death.

- **Serious adverse event or serious suspected adverse reaction**

An adverse event or suspected adverse reaction is considered "serious" if, in the view of either the investigator or sponsor, it results in any of the following outcomes: Death, a life-threatening adverse event, inpatient hospitalization or prolongation of existing hospitalization, a persistent or significant incapacity or substantial disruption of the ability to conduct normal life functions, or a congenital anomaly/birth defect. Important medical events that may not result in death, be life-threatening, or require hospitalization may be considered serious when, based upon appropriate medical judgment, they may jeopardize the patient or subject and may require medical or surgical intervention to prevent one of the outcomes listed in this definition. Examples of such medical events include allergic bronchospasm requiring intensive treatment in an emergency room or at home, blood dyscrasias or convulsions that do not result in inpatient hospitalization, or the development of drug dependency or drug abuse.

- **Suspected adverse reaction**

Any adverse event for which there is a reasonable possibility that the drug caused the adverse event. For the purposes of IND safety reporting, "reasonable possibility" means there is evidence to suggest a causal relationship between the drug and the adverse event. Suspected adverse reaction implies a lesser degree of certainty about causality than adverse reaction, which means any adverse event caused by a drug.

- **Unexpected adverse event or unexpected suspected adverse reaction**

An adverse event or suspected adverse reaction is considered "unexpected" if it is not listed in the investigator brochure or is not listed at the specificity or severity that has been observed; or, if

an investigator brochure is not required or available, is not consistent with the risk information described in the general investigational plan or elsewhere in the current application, as amended. For example, under this definition, hepatic necrosis would be unexpected (by virtue of greater severity) if the investigator brochure referred only to elevated hepatic enzymes or hepatitis. Similarly, cerebral thromboembolism and cerebral vasculitis would be unexpected (by virtue of greater specificity) if the investigator brochure listed only cerebral vascular accidents. "Unexpected," as used in this definition, also refers to adverse events or suspected adverse reactions that are mentioned in the investigator brochure as occurring with a class of drugs or as anticipated from the pharmacological properties of the drug, but are not specifically mentioned as occurring with the particular drug under investigation.

- **Severity Grading**

Adverse event severity will be evaluated utilizing the CTCAE v.5 criteria. If there is a question regarding severity grading or harmonization, the Sponsor or CRO should be contacted.

7.2.3. Reporting Adverse Events

- **Routine adverse events**

Adverse events that do not meet serious criteria (section 8.4) are considered routine adverse events. Routine adverse events are reported utilizing the appropriate eCRF page in the study database.

The investigator is reminded to comply with all institutional and local requirements as well as the requirements put forth by the IRB of record regarding reporting of routine adverse events.

- **Serious adverse events**

Any adverse event meeting serious criteria (section 8.2) occurring within the collection window must be reported following the instructions in Appendix 1 – Serious Adverse Event Reporting. **The timeframe for reporting** the event to the sponsor/CRO **is 1 business day** of the clinical investigator becoming aware of the initial event or clinically significant follow-up information.

The Investigator must provide a causality assessment and must sign and date all SAE Report Forms.

If supporting documentation is included in the submission (e.g., hospital reports, consultant reports, death certificates, autopsy reports, etc.), all protected health information must be redacted. When appropriate, additional written reports and supporting documentation will be provided by the site for further clarification and information.

The investigator is reminded to comply with all institutional and local requirements as well as the requirements put forth by the IRB of record regarding serious adverse events.

- **Adverse events leading to study discontinuation**

Participants who have withdrawn, or were withdrawn, from the study due to an adverse event (regardless of attribution) will be followed by the investigator until resolution or a new baseline, provided the participant consents to being followed.

- **Attribution to Investigational Medical Product and Study Procedure**

The relationship of [^{68}Ga]FAPI-46 and the PET scan procedure to an adverse event will be assessed by the Investigator utilizing the FDA's December 2012 guidance *Safety Reporting Requirements for INDs and BA/BE Studies*.

The investigator must note if there is a reasonable possibility there is evidence to suggest a causal relationship between [^{68}Ga]FAPI-46 and the adverse event and if there is evidence to suggest a causal relationship between the PET scan procedure and the adverse event.

Within the reporting requirement under 21 CFR§312.32(c)(1)(i), FDA makes clear the meaning of reasonable possibility by providing the following examples of types of evidence that would suggest a causal relationship between the drug and the adverse event.

- A single occurrence of an event that is uncommon and known to be strongly associated with drug exposure (e.g., angioedema, hepatic injury, Stevens-Johnson Syndrome)
- One or more occurrences of an event that is not commonly associated with drug exposure, but is otherwise uncommon in the population exposed to the drug (e.g., tendon rupture)
- An aggregate analysis of specific events observed in a clinical trial (such as known consequences of the underlying disease or condition under investigation or other events that those events occur more frequently in the drug treatment group than in a concurrent or historical control group

Although the sponsor is responsible for determining whether there is a reasonable possibility that the drug caused the adverse event, taking into consideration the investigator's assessment, the FDA requires the clinical investigator provide the sponsor with their assessment of causality for all serious adverse events.

- **Laboratory Abnormalities**

All abnormal laboratory results pre-categorized within the CTCAE v5 meeting grade 3 or 4 severity will be recorded and reported as AEs.

An abnormal laboratory result pre-categorized within the CTCAE v5 meeting grade 1 or 2 severity will be recorded and reported as an AE if the investigator deems the result clinically significant.

An abnormal laboratory result not pre-categorized within the CTCAE v5 (e.g., increased eosinophils, decreased creatinine) will not be captured as an adverse event unless deemed clinically significant by the investigator. The investigator must deem the result "mild," "moderate," "severe," or "life threatening," with the clinically significant notation.

- **Clinical Syndrome/Diagnosis vs. Symptomatic Components**

To the extent possible, individual symptomatic events (i.e., nausea, vomiting, laboratory abnormalities) observed during the course of the study should be reported as the overarching syndrome/diagnosis rather than the individual symptoms. For example, 'refeeding syndrome,' 'renal failure,' or 'hepatitis.' As appropriate, the laboratory abnormality itself (e.g., elevated creatinine) does not need to be recorded as an AE. Final determination of the appropriateness of recording (i.e. syndrome/diagnosis vs. symptom) is made by the clinical investigator.

- **Reporting Adverse Events to Participants**

The Sponsor will review all reported serious adverse events and determine the appropriateness of changes to the informed consent document. The sponsor will notify investigators if re-consenting subjects is mandatory for all participants or only for those currently undergoing investigational procedures. If the sponsor does not believe re-consenting is mandatory, the investigator may require re-consent for those all participants or just those subjects undergoing investigational procedures. Similarly, the sponsor will also defer to the IRB of record if it determines re-consent should be mandatory.

7.2.4. Documentation

Case Histories / Participant Binders

Adverse events should be recorded within the participant's case book / study binder compliant

with ALCOA. At minimum, information captured must include:

- Term, harmonized to CTCAE v. 5. Do not use a verbatim term or medical jargon – for example, use "white blood cell decreased," instead of *leukopenia* and "neutrophil count decreased," rather than *neutropenia*.
- Severity grade (as per CTCAE v. 5)
- Date, onset
- Date, end
- Outcome (e.g., without sequelae, with sequelae, ongoing)
- Attribution to study drug (unrelated, probable, definite)
- Attribution to the study procedure (i.e., PET/CT scan) (unrelated, probable, definite)

Attributions must be confirmed by the study's clinical investigator and documented by signature and date.

7.3. Unanticipated Problems

The phrase "unanticipated problems involving risks to subjects or others" is found but not defined in the HHS regulations at 45 CFR part 46. OHRP considers unanticipated problems, in general, to include any incident, experience, or outcome that meets **all** of the following criteria:

- Unexpected in terms of nature, severity, or frequency given (a) the research procedures that are described in the protocol-related documents, such as the Institutional Review Board (IRB)-approved research protocol and informed consent document; and (b) the characteristics of the participant population being studied;
- Related or possibly related to participation in the research ("possibly related" means there is a reasonable possibility that the incident, experience, or outcome may have been caused by the procedures involved in the research); and
- Suggests that the research places participants or others at a greater risk of harm (including physical, psychological, economic, or social harm) than was previously known or recognized.

OHRP recognizes that it may be difficult to determine whether a particular incident, experience, or outcome is unexpected and whether it is related or possibly related to participation in the research. OHRP notes that an incident, experience, or outcome that meets the three criteria above generally will warrant consideration of substantive changes in the research protocol or informed consent process/document or other corrective actions in order to protect the safety, welfare, or rights of subjects or others

Only a small subset of adverse events occurring in human subjects participating in research will meet these three criteria for an unanticipated problem.

Furthermore, there are other types of incidents, experiences, and outcomes that occur during the conduct of human subjects research that represent unanticipated problems but are not considered adverse events. For example, some unanticipated problems involve social or economic harm instead of the physical or psychological harm associated with adverse events. In other cases, unanticipated problems place subjects or others at increased risk of harm, but no harm occurs. Examples of unanticipated problems (UP) provided by OHRP:

- Clinical trial data are stored on a laptop computer without encryption, and the laptop computer is stolen from the investigator's car on the way home from work.
- As a result of a processing error by a pharmacy technician, a subject enrolled in a multicenter clinical trial receives a dose of an experimental agent that is 10-times higher than the dose dictated

by the IRB-approved protocol. While the dosing error increased the risk of toxic manifestations of the experimental agent, the subject experienced no detectable harm or adverse effect after an appropriate period of careful observation.

- Subjects with cancer are enrolled in a phase 2 clinical trial evaluating an investigational biologic product derived from human sera. After several subjects are enrolled and receive the investigational product, a study audit reveals that the investigational product administered to subjects was obtained from donors who were not appropriately screened and tested for several potential viral contaminants, including the human immunodeficiency virus and the hepatitis B virus.

7.3.1. Deviation from prescribed dose

In accordance with the NRC guidelines for diagnostic Nuclear Medicine, in the event of deviations from prescribed administered dose activity, the CRO/Sponsor should be contacted within 1 business day. This single intravenous (IV) injection should contain 5 mCi ± 2 mCi of [68Ga]FAPI-46 and a maximum mass dose of < 60 µg. The total volume administered will be between 1 and 10 mL. A protocol violation will be considered when [^{68}Ga]FAPI-46 dose administered is more than 20% of the prescribed activity.

If the dose deviation is reasonably associated with an adverse event (serious or non-serious), documentation for the adverse reaction must also be completed regardless of adverse reaction onset.

The investigator is reminded to comply with all institutional and local requirements as well as the requirements put forth by the IRB of record regarding protocol violations.

7.3.2. Pregnancy

A pregnancy occurring in a participant or in the partner of a participant during the course of the study (i.e. from study drug injection until end of study participation) is considered an unanticipated problem.

The investigator or site personnel must notify the Sponsor within 5 working days after the investigator or site personnel become aware of the pregnancy (see APPENDIX 1 – SERIOUS ADVERSE EVENT REPORTING for details). If an SAE occurs in conjunction with the pregnancy, then the reporting time frame for an SAE (within 1 business day) must be met.

The investigator is reminded to comply with all institutional and local requirements as well as the requirements put forth by the IRB of record regarding a pregnancy during trial participation.

7.3.3. Unanticipated Problem Reporting Adverse event as an unanticipated problem

An adverse event, or serious adverse event, also meeting the definition of an unanticipated problem will follow the adverse event reporting pathway.

7.3.4. Investigator-identified non-adverse event unanticipated problems

The investigator will provide information to the sponsor-investigator within 1 business day. The report will include the following information:

- Protocol identifying information: protocol title and number, investigator's name, and the participant study ID (if applicable)
- A detailed description of the event, incident, experience, or outcome;
- An explanation of the basis for determining that the event, incident, experience, or outcome represents an UP

- A description of corrective actions that have been taken or are proposed in response to the UP or proposed changes to the protocol or investigational plan

The investigator is reminded to comply with all institutional and local requirements as well as the requirements put forth by the IRB of record regarding unanticipated problems.

7.3.5. Reporting Unanticipated Problems to Participants

The sponsor will review all unanticipated problems and determine the appropriateness of

changes to the informed consent document. The sponsor will notify investigators if re-consenting subjects is mandatory for all participants or only for those currently undergoing investigational procedures.

The sponsor will defer to these IRBs if requested to change the risk section of the informed consent document. Similarly, the sponsor will also defer to the IRBs if it is determined re-consent should be mandatory.

8. STATISTICAL ANALYSIS

8.1. General

Descriptive statistics will be utilized for safety parameters. All patients will be accounted for. The study population for all analyses will be defined as all patients enrolled in the study who receive at least one dose of [^{68}Ga]FAPI-46. Patients who exit the study prior to receiving study medication may be replaced.

For this study, the primary efficacy endpoints are sensitivity and specificity, each of which will be evaluated with a hypothesis test:

H_0: Sensitivity (Specificity) ≤ Performance Goal (PG)

H_1: Sensitivity (Specificity) > Performance Goal (PG)

Where success is defined as rejecting the null hypothesis for the sensitivity hypothesis tests. The specificity test will have lower power given the lower number of samples that are likely to be negative from the histopathology sample.

The minimum sample size necessary to achieve desired power for sensitivity was computed for the hypothesis test above utilizing a one-sample binomial proportion test with a normal approximation, an upper one-sided significance level of 0.05, and the following parametric assumptions:

- Performance Goal (PG) = 0.75 for sensitivity and 0.70 for specificity
- True Sensitivity and Specificity = 0.90

Given the parametric assumptions above, 60 subjects will yield 92.7% power to demonstrate that the true sensitivity and specificity is greater than 0.75.

Additional exploratory data analyses will be conducted as deemed appropriate by the Sponsor.

8.2. Safety Analysis

All patients who receive any amount of [^{68}Ga]FAPI-46 will be included in the final summaries and listings of safety data.

Frequencies of patients experiencing at least one AE will be displayed by body system and preferred term according to MedDRA terminology. Detailed information collected for each AE will include: description of the event, duration, whether the AE was serious, intensity, relationship to study drug, action taken, clinical outcome, and whether or not it was a DLT. Intensity (severity) of the AEs will be graded according to the CTCAE v4.0. Emphasis in the analysis will be placed on AEs classified as dose limiting.

Summary tables will present the number of patients observed with AEs and corresponding percentages. The denominator used to calculate incidence percentages consists of patients

receiving at least one dose of study medication. Within each table, the AEs will be categorized by MedDRA body system and preferred term. Additional subcategories will be based on event intensity and relationship to study drug.

Deaths and other SAEs will be tabulated.

Vital signs will be summarized using descriptive statistics.

Summary tables will be prepared to examine the distribution of laboratory measures over time.

8.3. Efficacy Analysis

8.3.1. Primary Efficacy Analyses

The primary efficacy endpoints will compare the results from [^{68}Ga]FAPI-46 PET and histopathology. The table below displays the possible outcomes from the samples for each subject, with definitions for sensitivity and specificity.

		Histopathology	
		Positive	Negative
[^{68}Ga]FAPI-46 PET	Positive	A	B
	Negative	C	D
Total		A+C	B+D

Sensitivity= A / (A + C) Specificity = D / (B + D)

The primary analysis of sensitivity and specificity will be summarized using frequency counts and percentages as well as 95% asymptotic normal confidence intervals. Inference for the primary hypotheses will be conducted utilizing a one-sample binomial proportion test with a normal approximation, a one-sided upper significance level of 0.05, and assumed null proportions/performance goals of 0.75 for sensitivity. Success for the primary efficacy analyses will be defined as demonstrating that the sensitivity is statistically significantly greater than their performance goal.

Accuracy of [^{68}Ga]FAPI-46 PET for the detection of FAP expressing cells compared to histopathology, defined as

Accuracy = (A + D) / (A + B + C + D)

will be presented with a 95% confidence interval.

8.3.2. Secondary Efficacy Analyses

Association between SUV_{max} from [^{68}GaFAPI-46 PET on cross sectional imaging and FAP expression from IHC will be summarized by correlation coefficients with 95% confidence intervals.

8.4. Pharmacodynamic Analysis

Samples of serum for CA 19-9 (or CA125, CEA if non-secretors for pancreas cancer), CEA and CA125 as applicable, any tumor marker appropriate to the given cancer or that is known to be elevated in a given patient will be collected and processed as per the instructions provided in the respective lab manuals. Serum tumor biomarkers levels will also be performed as per the instructions provided in the respective lab manuals.

8.5. Tumor Response

All tumor responses will be summarized and reported by RECIST criteria (see Appendix). In addition, change in tumor measurements not meeting RECIST criteria will be displayed in order to detect subclinical biologic activity of this new drug.

8.6. Interim Analysis

Not applicable

8.7. Sample Size

Up to 60 patients will be enrolled in the study. See SAP for full description of assumptions and analytical plan.

9. SUPPORTING DOCUMENTATION AND OPERATIONAL CONSIDERATIONS

9.1. Regulatory, Ethical, And Study Oversight Considerations

9.1.1. Informed Consent Process

Informed consent and other documents provided to participant

Consent forms describing in detail the study agent, study procedures, and risks will be given to the participant and written documentation of informed consent is required prior to starting research procedures. None of the elements of consent outlined in 21CFR§50.25 will be waived.

Authorization of use and disclosure of protected health information (PHI) must be obtained from each participant prior to performing any study-specific screening evaluations. The authorization for use and disclosure of PHI must contain the elements required by 45 CFR 164.508(b) for valid authorizations.

A copy of the signed document will be provided to the participant. The original signed informed consent document will be maintained in the participant's case history (i.e. study binder). Written documentation of the consent process, signed and dated by the person who obtained consent, must be completed and maintained in the participant's case history (i.e., study binder).

Written documentation of the consent process must be completed for any reconsent, including the reason for the reconsent.

9.1.2. Study Discontinuation and Closure

This study may be temporarily suspended or prematurely terminated if there is sufficient reasonable cause. Written notification, documenting the reason for study suspension or termination, will be provided by the Sponsor or designee. If the study is prematurely terminated or suspended, the Investigator will promptly inform participants actively undergoing study assessment as well as the IRB of record. The Investigator will provide the reason(s) for the

termination or suspension to the IRB of record and should, as allowed by the IRB, provide the same information to the participant as appropriate.

Study participants will be contacted, as applicable, and be informed of changes to study visit schedule.

Circumstances that may warrant termination or suspension include, but are not limited to:

- Determination of unexpected, significant, or unacceptable risk to participants
- Demonstration of efficacy that would warrant stopping
- Insufficient compliance to protocol requirements
- Data that are not sufficiently complete and/or evaluable
- Determination that the primary endpoint has been met
- Determination of futility

See also Protocol Section 7.

9.1.3. Participant and Data Confidentiality Data

All information regarding the nature of the proposed investigation provided by the Sponsor or Study Monitor to the Investigator (with the exception of information required by law or regulations to be disclosed to the IRB, the patient, or the appropriate regulatory authority) must be kept in confidence by the Investigator.

9.1.4. Participant Confidentiality

Participant confidentiality is strictly held in trust by the participating investigators, their staff, and the sponsor(s) and their agents. This confidentiality is extended to cover testing of biological samples and genetic tests in addition to the clinical information relating to participants. Therefore, the study protocol, documentation, data, and all other information generated will be held in strict confidence. No information concerning the study or the data will be released to any unauthorized third party without prior written approval of the sponsor.

The study monitor, other authorized representatives of the sponsor, the Sponsor, or representatives of the IRB of record may inspect all documents and records required to be maintained by the investigator, including but not limited to, medical records (office, clinic, or hospital) and pharmacy records for the participants in this study. The clinical study site will permit access to such records.

The study participant's contact information will be securely stored at each clinical site for internal use during the study. At the end of the study, all records will continue to be kept in a secure location for as long a period as dictated by IRB and Institutional regulations. Study participant research data, which is for purposes of statistical analysis and scientific reporting, will be reported using the study's electronic database, which is 21CFR§11 compliant. **This will not include the participant's contact or identifying information** as defined by 45CFR§164.514(b)(2). Rather, individual participants and their research data will be identified by a unique study identification number. The study data entry and study management systems used by clinical sites and by research staff will be secured and password protected.

Of note, **the participant's actual date of birth must not be used for any study documentation**. The participant's date of birth must be "reset" to 01 of the appropriate month. For example, a birthdate of 24 December 2000 would be 01 December 2000.

9.1.5. Research Use of Stored Human Specimens

Tissue samples will be obtained and stored as described in the Laboratory Manual.

9.1.6. Study Governance, Key Roles, and Accreditations

- **<u>Study Project Manager</u>**

Sherly Mosessian, Ph.D

Vice President Clinical and Regulatory Affairs

SOFIE

- **Study Medical Monitor**

Elcin Zan, M.D

Neuroradiologist

9.1.7. Site Investigator Information

The investigator will provide appropriate documentation of qualification as outlined in form FDA 1572.

The investigator is responsible to ensure the site conducts this clinical trial is conducted in full conformity with Regulations for the Protection of Human Subjects of Research codified in 45 CFR Part 46, 21 CFR Part 50, 21 CFR Part 56, and 21 CFR Part 312.

9.1.8. Delegation of Site Investigator Responsibilities

The Investigator should ensure that all persons involved in the conduct of the study are informed about the protocol, protocol amendments, study procedures, and study related duties.

9.1.9. Laboratory Accreditations

Clinical laboratories to be used for the purposes of hematology, serum chemistry, tumor markers, and pathologic analysis within this protocol must be listed on the Investigator's form FDA 1572. The form should be updated if additional clinical laboratories are utilized.

Licensure/accreditations and reference values and/or normal ranges for the test results must be provided to the Sponsor or designee.

9.1.10. Safety Oversight

Safety oversight will be under the direction of a Data and Safety Monitoring Committee composed of individuals with the appropriate expertise.

9.1.11. Clinical Monitoring

"Effective monitoring of clinical investigations by sponsors is critical to the protection of human subjects and the conduct of high-quality studies. Sponsors of clinical investigations involving human drugs, biological products, medical devices, and combinations thereof are required to provide oversight to ensure adequate protection of the rights, welfare, and safety of human subjects and the quality of the clinical trial data submitted to FDA. FDA's regulations require sponsors to monitor the conduct and progress of their clinical investigations. The

regulations are not specific about how sponsors are to conduct such monitoring and are therefore compatible with a range of approaches to monitoring that will vary depending on multiple factors." Page 2 of FDA's *Guidance for Industry: Oversight of Clinical Investigations — A Risk-Based Approach to Monitoring* (October 2013).

A risk based monitoring approach will be employed for this clinical trial.

- Centralized monitoring, utilizing EDC data analysis for outliers and inconsistent data patterns.
- Remote monitoring visits. Preferably, RMV will utilize electronic source (i.e., access to electronic health records, HIPAA/HITECH approved source data review) but surrogate source may be utilized as deemed appropriate.
- Directed on-site monitoring. These will be performed as determined by the study sponsor or designee. All sites will undergo at least one on-site monitoring visit to determine compliance to protocol and investigational plan, adequacy of documentation practices and good clinical practice.

Monitoring reports must be filed with the IRB of record unless prohibited by policy. If prohibited, a copy of the policy must be provided for both the ISF and trial master file.

9.1.12. Quality Assurance and Quality Control

This section will briefly describe the plans for quality management, the system for assessing the quality of processes within a system. Quality management encompasses quality assurance (QA) and quality control (QC) .

Each site, both clinical and laboratory, should have SOPs for quality management that describe:

- How data and biological specimens (when applicable) will be evaluated for compliance with the protocol, ethical standards, regulatory compliance, and accuracy in relation to source documents.
- The documents to be reviewed (e.g., CRFs, clinic notes, product accountability records, specimen tracking logs, questionnaires, audio or video recordings), who is responsible, and the frequency for reviews.
- Who will be responsible for addressing QA issues (e.g., correcting procedures that are not in compliance with protocol) and QC issues (e.g., correcting errors in data entry).
- Staff training methods and how such training will be tracked.

If applicable, calibration exercises conducted prior to and during the study to train examiners and maintain acceptable intra- and inter-examiner agreement.

Regular monitoring and an independent audit, if conducted, must be performed according to ICH GCP.

9.1.13. Data Handling and Record Keeping Data collection & management

Data collection is the responsibility of the clinical trial staff at the site under the supervision of the investigator. The investigator is responsible for ensuring the accuracy, completeness, legibility, and timeliness of the data reported. All source documents should be completed in a neat, legible manner to ensure accurate interpretation of data. An indelible ink that will ensure clarity of reproduced copies is required. When making changes or corrections, cross out the original entry with a single line, and initial and date the change. All source documents must meet the FDA standard of ALCOA. DO NOT ERASE, OVERWRITE, OR USE CORRECTION FLUID OR TAPE ON THE ORIGINAL.

Data reported in the eCRF derived from source documents should be consistent with the source documents or the discrepancies should be explained and captured in a progress note and maintained in the participant's official electronic study record. Clinical data (including AEs, concomitant medications, and expected adverse reactions data) and clinical laboratory data will be entered into a 21 CFR Part 11-compliant data capture system provided by the CRO/Sponsor. The data system includes password protection and internal quality checks, such as automatic range checks, to identify data that appear inconsistent, incomplete, or inaccurate. Clinical data will be entered directly from the source documents.

9.1.14. Study document retention

Federal law at 21 CFR§312.62 mandates records required by 21CFR§312 (e.g., records of drug receipt and disposition, signed and dated consent forms, medical records including progress notes of the physician, the individual's hospital chart(s), and the nurses' notes) must be retained by the investigator for at least 2 years following the date a marketing application is approved for the drug for the indication for which it is being investigated; or, if no application is to be filed or if the application is not approved for such indication, until 2 years after the investigation is discontinued and FDA is notified.

No study documents will be destroyed or moved to a new location without prior written approval from the Sponsor.

No records will be destroyed without the written consent of the sponsor. It is the responsibility of the sponsor to inform the investigator when these documents no longer need to be retained.

These documents may need to be retained for a longer period, if required by local regulations.

9.1.15. Protocol Deviations

A protocol deviation is any noncompliance with the protocol, GCP, or procedure manuals (e.g., imaging manual, laboratory manual). The noncompliance may be either on the part of the participant, the investigator, or the study site staff.

As a result of deviations, corrective actions are to be developed by the site and implemented promptly.

These practices are consistent with ICH E6(R2):

- 4.5 Compliance with Protocol, sections 4.5.1, 4.5.2, and 4.5.3
- 5.1 Quality Assurance and Quality Control, section 5.1.1
- 5.20 Noncompliance, sections 5.20.1, and 5.20.2.

It is the responsibility of the site to use continuous vigilance to identify and record deviations contemporaneously.

All deviations must be addressed in study source documents and reported to the study monitor.

Protocol deviations must be sent to the IRB of record per their guidelines. If the IRB of record

has a policy prohibiting submission of deviations, a copy of this policy must be filed in both the ISF and trial master file.

The investigator and study staff are responsible for knowing and adhering to their IRB requirements.

9.1.16. Forward planned deviations

The Investigator is not allowed to institute changes to the protocol or investigational plan except when necessary to protect the safety, rights, or welfare of subjects.

- If a forward-planned deviation occurs to protect the safety, rights or welfare of subjects, the Investigator or designee must notify the Sponsor or Designee in writing within 1 business day. The deviation must specify the perceived risk and if, in the investigator's opinion, it impacted the study's safety or outcome data. Lastly, the Investigator or designee must provide an opinion if this change should be made for all participants or if it was a single occurrence.
- The notice of deviation must be filed with the IRB in accordance with its policy.
- The Sponsor or designee will provide a formal determination regarding the deviation. If the Sponsor or designee concurs with the deviation, a protocol clarification letter and/or protocol amendment will be initiated. If the Sponsor or designee does not concur with the deviation, the determination will provide recommendations for similar situations for the participant's safety, rights, or welfare to be maintained while actively participating in the protocol.

9.1.17. Eligibility deviations

- Deviations from eligibility criteria are considered protocol violations
- These violations must be reported to the Sponsor or designee upon identification as well as to the IRB of record.
- The site is placed on accrual hold until a root cause analysis and corrective action plan is provided to the Sponsor or designee for review and it is approved.

9.1.18. Major deviations

Major deviations are those that are considered to negatively participant safety, rights or welfare of a participant or that may negatively impact study results. These must be reported to the Sponsor or designee within 5 business days and must include a root cause analysis as well as a corrective action plan. Major deviations include, but are not limited to:

- Inadequate informed consent, which is defined as failing to follow the stipulations of the IRB of record, failing to adequately document informed consent was obtained prior to undertaking research-only procedures, filing to obtain informed consent as per the IRB of record, or failing to use the appropriate version of the informed consent
- Failing to submit an amendment (e.g., protocol, investigator's brochure) or revised informed consent document to the institutional review board of record within 60 days of receipt
- Falsification, fabrication, or other scientific misconduct
- Individual prescribing investigational medical product who is not authorized to do so
- Investigational agent administered to the incorrect patient
- Overdose of investigational medical product (as defined by Section 9.1)

- Failing to obtaining continuing review (i.e., lapse of IRB approval)
- Failing to review suspected unexpected serious adverse reaction (SUSAR) reports

9.1.19. Publication and Data Sharing Publication(s)

The Sponsor recognizes the importance of and will fulfill its obligation for the disclosure of clinical research findings, timely and in appropriate forums. Any disclosure of results from the Sponsor’s clinical trials will be submitted to the Sponsor for review to assure accuracy of information and to protect proprietary documents and materials. This review will not be unreasonably withheld and provided in writing within 30 days of receipt. Clinical trials conducted by more than one investigational site, multi-center studies, will be reported jointly with all patients represented in the analyses.

9.1.20. Disclosure of Data

All information obtained as a result of this study or during the conduct of this study will be regarded as confidential. Disclosures (i.e., any release of information to any third party not noted herein) of any, not previously known to be public, information and/or results of the investigation for publication or by capsules or poster presentation shall not be made earlier than 30 days after submission of the proposed material to the sponsor for inspection, unless the sponsor consents to earlier disclosure. The Investigator will take appropriate cognizance of the sponsor's suggestions before disclosure for publication or presentation consistent with protection of the sponsor's right to its confidential data.

9.1.21. Conflict of Interest and Financial Disclosure

The independence of this study from any actual or perceived influence is critical. Any actual conflict of interest of persons who have a role in the design, conduct, analysis, publication, or any aspect of this trial will be disclosed and managed.

The Investigator shall provide the sponsor with sufficient accurate financial information to allow the Sponsor or designated Applicant to submit complete and accurate certification or disclosure statements as required under 21 CFR§54.

As per 21CFR§312.64(d) the Site Investigator will provide financial information for:

- The 1572 investigator(s)
- The spouse and/or each dependent child of each 1572 investigator
- 1572 sub investigator(s) directly involved in the treatment or evaluation of research subjects
- The spouse and/or each dependent child of each sub investigator

The Investigator shall promptly update this information if any relevant changes occur during the course of the investigation and for 1 year following the completion of the study.

Investigators are reminded to file the appropriate documentation with their institution for review and management of conflict of interest.

9.2. Source Documentation

The Investigator must make study data accessible to the Sponsor, to other authorized representatives of the Sponsor, and to the appropriate regulatory authority inspectors. The eCRF for each patient will

be checked against source documents at the study site by the Study Monitor.

Each participating site will maintain appropriate medical and research records for this trial, in compliance with federal code at 21CFR§312 as well as applicable regulatory and institutional requirements for the protection of confidentiality of participants. As part of participating in this study, each site will permit authorized representatives of the Sponsor and regulatory agencies to examine (and when permitted by applicable law, to copy) clinical records for the purposes of quality assurance reviews, audits, and evaluation of the study safety, progress, and data validity.

Source data are all information, original records of clinical findings, observations, or other activities in a clinical trial necessary for the reconstruction and evaluation of the trial. Examples of these original documents and data records include, but are not limited to, hospital records, clinical and office charts, laboratory notes, memoranda, evaluation checklists, pharmacy dispensing records, recorded data from automated instruments, copies or transcriptions certified after verification as being accurate and complete, microfiches, photographic negatives, microfilm or magnetic media, x-rays, and participant files and records kept at the pharmacy, at the laboratories, and medico-technical departments involved in the clinical trial.

It is not acceptable to use printed electronic case report form(s) as source documents or to structure source documents exclusively from the data required for the study database. This creates a tunnel effect when evaluating participants for safety and/or outcomes.

It is not acceptable for the case report form or shadow chart to be the only record of a patient's participation in this clinical trial. This is to ensure that anyone who would access the patient medical record has adequate knowledge that the patient is participating in a clinical trial of an investigational medical product.

9.2.1. Investigator's Site Files (Regulatory Binder)

The Investigator will maintain an investigator site file (ISF; regulatory binder) containing essential documentation identified by the Sponsor or designee. Essential documentation will be outlined through a table of contents provided by the Sponsor/designee as well as applicable documentation (i.e., monitor visit logs, screening logs, training logs, delegation logs, authorized prescriber log, sample SAE reporting forms, sample pregnancy reporting forms).

Copies of all reports to and correspondence with and from the IRB/EC will be provided to the Sponsor or their designee.

If not maintained through electronic software, the Investigator must maintain a correspondence file between the Investigator's site and the IRB of record. This correspondence must be made available for monitoring upon request.

The ISF documentation will align with the trial master file maintained by the sponsor or designee.

9.2.2. Required Documentation for Site Activation

The following source documentation must be collected from the Investigator and reviewed prior to shipment of the investigational medical product (study drug) to the Investigator.

Wet-ink documents. A scanned version is acceptable for initial review; however, wet-ink must be maintained as soon as feasible. The wet-ink must be compared against the scanned version for accuracy and then stored in the trial master file. If a discrepancy is noted, the study site is placed on hold until resolution. Each site ISF must have a documentation as to the location of the source. Electronic signatures are not acceptable for these documents.

- FDA form 1572
- Protocol signature page
- Investigator's brochure signature page

Ethics documents. The following documents must be provided to the sponsor or designee. Wet-ink copies are not required.

- IRB letter documenting favorable outcome, stipulations regarding consent, version of protocol reviewed, and version of investigator's brochure reviewed. If the IRB letter does not document stipulations of consent or protocol version, a copy of the IRB application with an attestation by appropriately delegated site personnel can be used.
- Stamped IRB-approved informed consent document, and stamped patient-facing materials must be provided. If the IRB does not utilize approval stamps for the consent and/or patient-facing materials, a copy of the policy or note to file must be filed in both the ISF and trial master file.
- A favorable site initiation report must be filed by the designated personnel. If site enrollment is to begin on the same day as the site initiation visit, the report must clearly state the time (24hh) the site was allowed to initiate enrollment and then be faxed or emailed to the Sponsor or designee. A fax receipt, or copy of the sent email, must be retained with appropriate time stamps. Best practice is for the Sponsor or designee to confirm receipt of the SIV report.

Operational documentation. The following information is required for operationalization of the clinical trial and does not impact enrollment:

- The sponsor or designee must have copies of the appropriate federal, state, and local documentation regarding the receipt of radioactive materials by the Investigator's site
- Shipping address for investigational medical product as well as point-of-contact information.

9.3. INVESTIGATOR AGREEMENT

I have read the foregoing protocol *"A Phase 2,Multicenter, Single Arm, Open Label, Non-Randomized Study of [^{68}Ga]FAPI-46 PET in patients with pancreatic ductal adenocarcinoma (PDAC) with resectable or borderline resectable disease considered potential candidates for surgery "* and agree to conduct the study as described therein.

I will conduct this protocol as outlined therein and will make a reasonable effort to complete the study within the time designated. I will provide copies of the protocol and access to all information furnished by SOFIE Biosciences to study personnel under my supervision. I will discuss this material with them to ensure they are fully informed about the drug and the study.

I understand that the study may be terminated or enrollment suspended at any time by SOFIE Biosciences, Inc., with or without cause, or by me if it becomes necessary to protect the best interests of the study patients.

____

Investigator's Name, printed

____ ____________________

Investigator's Signature date (dd/mm/yyyy)

10. REFERENCES

"Cancer Facts & Figures 2021," American Cancer Society, Atlanta, 2021.

C. Kratochwil *et al.*, "68Ga-FAPI PET/CT: Tracer Uptake in 28 Different Kinds of Cancer," *J. Nucl. Med.*, vol. 60, no. 6, pp. 801–805, 2019, doi: 10.2967/jnumed.119.227967.

T. Lindner *et al.*, "Development of Quinoline-Based Theranostic Ligands for the Targeting of Fibroblast Activation Protein," *J. Nucl. Med.*, vol. 59, no. 9, pp. 1415–1422, 2018, doi: 10.2967/jnumed.118.210443.

A. Loktev *et al.*, "Development of Fibroblast Activation Protein-Targeted Radiotracers with Improved Tumor Retention," *J. Nucl. Med.*, vol. 60, no. 10, pp. 1421–1429, 2019, doi: 10.2967/jnumed.118.224469.

A. Loktev *et al.*, "A Tumor-Imaging Method Targeting Cancer-Associated Fibroblasts," *J. Nucl. Med.*, vol. 59, no. 9, pp. 1423–1429, 2018, doi: 10.2967/jnumed.118.210435.

Meyer *et al.*, "Radiation dosimetry and biodistribution of ^{68}Ga-FAPI-46 PET imaging in cancer patients," *J Nucl Med*, p. jnumed.119.236786, Dec. 2019, doi: 10.2967/jnumed.119.236786.

M. Röhrich *et al.*, "Impact of ^{68}Ga-FAPI-PET/CT imaging on the therapeutic management of primary and recurrent pancreatic ductal adenocarcinomas," *J Nucl Med*, p. jnumed.120.253062, Oct. 2020, doi: 10.2967/jnumed.120.253062.

Wang Z, Chen JQ, Liu J-L et al. FDG-PET in the diagnosis, staging and prognosis of pancreatic carcinoma: A meta-analysis. Wold J Gastroenterol. 2013 Aug7; 19(29): 4808-4817.

T. Watabe *et al.*, "Theranostics Targeting Fibroblast Activation Protein in the Tumor Stroma: ^{64}Cu- and ^{225}Ac-Labeled FAPI-04 in Pancreatic Cancer Xenograft Mouse Models," *J Nucl Med*, vol. 61, no. 4, pp. 563–569, Apr. 2020, doi: 10.2967/jnumed.119.233122.

11. APPENDICES

11.1. Appendix 1: Serious Adverse Event Reporting

CONTACT THE SOFIE BIOSCIENCES, INC. MEDICAL MONITOR WITHIN 24 HOURS OF LEARNING OF ANY SERIOUS ADVERSE EVENT. IF THE SERIOUS ADVERSE EVENT IS FATAL OR LIFE THREATENING, THE SPONSOR MUST BE INFORMED IMMEDIATELY.

For reporting of serious adverse events, Investigators must enter the SAE report form into database within 24 hours to the following:
To discuss SAE with the Medical Monitor, contact <TBD>> at the numbers provided below: Name: TBD Office #: TBD Cell #: TBD Email: TBD
All reports of SAEs should be entered into the database within one working day. Follow-up information to SAEs must also be entered into the database within 24 hours as it becomes known **to the investigator.**

11.2. Appendix 2: Eastern Cooperative Group (ECOG) Performance Status Scale

**ECOG PERFORMANCE STATUS***	
**Grade**	**ECOG**
0	Fully active, able to carry on all pre-disease performance without restriction
1	Restricted in physically strenuous activity but ambulatory and able to carry out work of a light or sedentary nature, e.g., light housework, office work
2	Ambulatory and capable of all selfcare but unable to carry out any work activities. Up and about more than 50% of waking hours
3	Capable of only limited selfcare, confined to bed or chair more than 50% of waking hours
4	Completely disabled. Cannot carry on any selfcare. Totally confined to bed or chair
5	Dead

* From ECOG, Robert Comis, MD, Group Chair

Oken MM., Creech RH, Tormey DC, Horton J, Davis TE, McFadden ET, Carbone PP. Toxicity and Response Criteria of The Eastern Cooperative Oncology Group. Am J Clin Oncol 1982; 5:649-655.

11.3. Appendix 3: Response Evaluation Criteria in Solid Tumors (RECIST 1.1) QUICK REFERENCE

11.3.1. Eligibility

Only patients with measurable disease at baseline should be included in protocols where objective tumor response is the primary endpoint. Measurable disease is defined as the presence of at least one measurable lesion.

11.3.2. Methods of Assessment

The same method of assessment and the same technique should be used to characterize each identified and reported lesion at baseline and during follow-up.

- CT is the best currently available and reproducible method to measure lesions selected for response assessment. MRI is also acceptable in certain situations (e.g., for body scans but not for lung).
- Lesions on a chest X-ray may be considered measurable lesions if they are clearly defined and surrounded by aerated lung. However, CT is preferable.
- Clinical lesions will only be considered measurable when they are superficial and ≥10 mm in diameter as assessed using calipers. For the case of skin lesions, documentation by color photography, including a ruler to estimate the size of the lesion, is recommended.
- Ultrasound (US) should not be used to measure tumor lesions.
- Tumor markers alone cannot be used to assess response. If markers are initially above the upper normal limit, they must normalize for a patient to be considered in complete response.
- Cytology and histology can be used in rare cases (e.g., for evaluation of residual masses to differentiate between Partial Response [PR] and Complete Response [CR] or evaluation of new or enlarging effusions to differentiate between Progressive Disease [PD] and Response/Stable Disease [SD]).
- Use of endoscopy and laparoscopy is not advised. However, they can be used to confirm complete pathological response.

11.3.3. Baseline Disease Assessment

All baseline evaluations should be performed as closely as possible to the beginning of treatment and never more than 4 weeks before the beginning of the treatment.

Measurable Lesions - Must be accurately measured in at least one dimension (longest diameter in the plane of measurement is to be recorded) with a minimum size of:

- 10 mm by CT scan (CT scan slice thickness no greater than 5 mm; when CT scans have slice thickness >5 mm, the minimum size should be twice the slice thickness).
- 10 mm caliper measurement by clinical exam (lesions which cannot be accurately measured with calipers should be recorded as non-measurable).
- 20 mm by chest X-ray.
- Malignant lymph nodes
  - o To be considered pathologically enlarged and measurable, a lymph node must be ≥15 mm in short axis when assessed by CT scan (CT scan slice thickness is recommended to be no greater than 5 mm). At baseline and in follow-up, only

the short axis will be measured and followed.

- Lytic bone lesions or mixed lytic-blastic lesions with identifiable soft tissue components that can be evaluated by cross-sectional imaging techniques such as CT or MRI can be considered measurable if the soft tissue component meets the definition of measurability described above.
- 'Cystic lesions' thought to represent cystic metastases can be considered measurable if they meet the definition of measurability described above. However, if non-cystic lesions are present in the same patient, these are preferred for selection as target lesions.

Non-Measurable Lesions - Non-measurable lesions are all other lesions, including small lesions (longest diameter <10 mm or pathological lymph nodes with 10 to <15 mm short axis), as well as truly non-measurable lesions. Lesions considered truly non-measurable include: leptomeningeal disease, ascites, pleural or pericardial effusion, inflammatory breast disease, lymphangitic involvement of skin or lung, abdominal masses/abdominal organomegaly identified by physical exam that is not measurable by reproducible imaging techniques.

Blastic bone lesions are non-measurable.

Lesions with prior local treatment, such as those situated in a previously irradiated area or in an area subjected to other loco-regional therapy, are usually not considered measurable unless there has been demonstrated progression in the lesion. Study protocols should detail the conditions under which such lesions would be considered measurable.

11.3.4. Target Lesions

- All measurable lesions up to a maximum of two lesions per organ and five lesions in total, representative of all involved organs, should be identified as target lesions and recorded and measured at baseline.
- Target lesions should be selected on the basis of their size (lesions with the longest diameter) and be representative of all involved organs, as well as their suitability for reproducible repeated measurements.
- All measurements should be recorded in metric notation using calipers if clinically assessed.

A sum of the diameters (longest for non-nodal lesions, short axis for nodal lesions) for all target lesions will be calculated and reported as the baseline sum diameters, which will be used as reference to further characterize any objective tumor regression in the measurable dimension of the disease. If lymph nodes are to be included in the sum, only the short axis will contribute.

11.3.5. Non-Target Lesions

All lesions (or sites of disease) not identified as target lesions, including pathological lymph nodes and all non-measurable lesions, should be identified as non-target lesions and be recorded at baseline. Measurements of these lesions are not required and they should be followed as 'present', 'absent' or in rare cases, 'unequivocal progression'.

11.3.6. Response Criteria Evaluation of target lesions

Complete Response (CR)	Disappearance of all target lesions. Any pathological lymph nodes (whether target or non-target) must have reduction in short axis to <10 mm.
Partial Response (PR)	At least a 30% decrease in the sum of the diameters of target lesions, taking as reference the baseline sum of diameters
Progressive Disease (PD)	At least a 20% increase in the sum of the LD of target lesions, taking as reference the smallest sum on study (this may include the baseline sum). The sum must also demonstrate an absolute increase of at least 5 mm
Stable Disease (SD)	Neither sufficient shrinkage to qualify for PR nor sufficient increase to qualify for PD

<u>Special notes on the assessment of target lesions</u>

- Lymph nodes identified as target lesions should always have the actual short axis measurement recorded even if the nodes regress to below 10 mm on study. When lymph nodes are included as target lesions, the ‘sum’ of lesions may not be zero even if complete response criteria are met since a normal lymph node is defined as having a short axis of <10 mm.
- Target lesions that become ‘too small to measure’. While on study, all lesions (nodal and non- nodal) recorded at baseline should have their actual measurements recorded at each subsequent evaluation, even when very small. However, sometimes lesions or lymph nodes become so faint on a CT scan that the radiologist may not feel comfortable assigning an exact measure and may report them as being ‘too small to measure’, in which case a default value of 5 mm should be assigned.
- Lesions that split or coalesce on treatment. When non-nodal lesions ‘fragment’, the longest diameters of the fragmented portions should be added together to calculate the target lesion sum. Similarly, as lesions coalesce, a plane between them may be maintained that would aid in obtaining maximal diameter measurements of each individual lesion. If the lesions have truly coalesced such that they are no longer separable, the vector of the longest diameter in this instance should be the maximal longest diameter for the ‘coalesced lesion’.

11.3.7. Evaluation of non-target lesions

Complete Response (CR)	Disappearance of all non-target lesions and normalization of tumor marker levels. All lymph nodes must be non-pathological in size (< 10 mm short axis)
Non-CR/Non-PD	Persistence of one or more non-target lesion(s) or/and maintenance of tumor marker level above the normal limits
Progressive Disease (PD)	Unequivocal progression of existing non-target lesions.<br>When patient has measurable disease. To achieve ‘unequivocal progression’ on the basis of the non-target disease, there must be an overall level of substantial worsening in non-target disease such that, even in presence of SD or PR in target disease, the overall tumor burden has increased sufficiently to merit discontinuation of therapy. A modest ‘increase’ in the size of one or more non-target lesions is usually not sufficient to qualify for unequivocal progression status<br>• When patient has only non-measurable disease. There is no measurable disease assessment to factor into the interpretation of an increase in non- measurable disease burden. Because worsening in non-target disease cannot be easily quantified, a useful test that can be applied is to consider if the increase in overall disease burden based on change in non-measurable disease is comparable in magnitude to the increase that would be required to declare PD for measurable disease. Examples include an increase in apleural effusion from ‘trace’ to ‘large’ or an increase in lymphangitic disease from localized to

	widespread.

New Lesions

The appearance of new malignant lesions denotes disease progression:

- The finding of a new lesion should be unequivocal (i.e., not attributable to differences in scanning technique, change in imaging modality or findings thought to represent something other than tumor, especially when the patient's baseline lesions show partial or complete response).
- If a new lesion is equivocal, for example because of its small size, continued therapy and follow- up evaluation will clarify if it represents truly new disease. If repeat scans confirm there is definitely a new lesion, then progression should be declared using the date of the initial scan.
- A lesion identified on a follow-up study in an anatomical location that was not scanned at baseline is considered a new lesion and disease progression.

11.3.8. [^{18}F]FDG-PET

It is sometimes reasonable to incorporate the use of [^{18}F]FDG-PET scanning to complement CT in assessment of progression (particularly possible 'new' disease). New lesions on the basis of [^{18}F]FDG-PET imaging can be identified according to the following algorithm:

Negative [^{18}F]FDG-PET at baseline, with a positive [^{18}F]FDG-PET at follow-up is PD based on a new lesion.

No [^{18}F]FDG-PET at baseline and a positive [^{18}F]FDG-PET at follow-up:

- If the positive [^{18}F]FDG-PET at follow-up corresponds to a new site of disease confirmed by CT, this is PD.
- If the positive [^{18}F]FDG-PET at follow-up is not confirmed as a new site of disease on CT, additional follow-up CT scans are needed to determine if there is truly progression occurring at that site (if so, the date of PD will be the date of the initial abnormal [^{18}F]FDG-PET scan).
- If the positive [^{18}F]FDG-PET at follow-up corresponds to a pre-existing site of disease on CT that is not progressing on the basis of the anatomic images, this is not PD.

11.3.9. Time Point response

A summary of the overall response status calculation at each time point for patients who have measurable disease at baseline is presented in Table A1 below.

11.3.10. Table A1. Time point response: Patients with target (+/–non-target) disease

Target lesions	Non-Target lesions	New Lesions	Overall response
CR	CR	No	CR
CR	Non-CR/non-PD	No	PR
CR	NE	No	PR
PR	Non-PD/or not all evaluated	No	PR
SD	Non-PD/or not all evaluated	No	SD
Not all evaluated	Non-PD	No	NE
PD	Any	Yes or No	PD
Any	PD	Yes or No	PD

Any	Any	Yes	PD

CR = complete response; PR = partial response; SD = stable disease; PD = progressive disease; NE = inevaluable.

When patients have non-measurable (therefore non-target) disease only, Table A2 is to be used.

11.3.11. Table A2. Time point response: Patients with non-target disease

Non-Target lesions	New Lesions	Overall response
CR	No	CR
Non-CR/non-PD	No	Non-CR/non-PD[1]
Not all evaluated	No	NE
PD	Yes or No	PD
Unequivocal PD	Yes or No	PD
Any	Yes	PD

CR = complete response; PR = partial response; SD = stable disease; PD = progressive disease; NE = inevaluable.

[1] Non-CR / non-PD is preferred over 'Stable Disease' for non-target disease since SD is increasingly used as an endpoint for assessment of efficacy in some trials. To assign this category when no lesions can be measured is not advised

11.3.12. Confirmation

In non-randomized trials where response is the primary endpoint, confirmation of PR and CR is required to ensure responses identified are not the result of measurement error. This will also permit appropriate interpretation of results in the context of historical data where response has traditionally required confirmation in such trials.

However, in all other circumstances, (i.e., in randomized phase 1I or III trials or studies where stable disease or progression are the primary endpoints), confirmation of response is not required since it will not add value to the interpretation of trial results. However, elimination of the requirement for response confirmation may increase the importance of central review to protect against bias, in particular in studies, which are not blinded.

In the case of SD, measurements must have met the SD criteria at least once after study entry at a minimum interval (in general not less than 6–8 weeks) that is defined in the study protocol.

11.3.13. Missing Assessments and Inevaluable Designation

When no imaging/measurement is done at all at a particular time point, the patient is not evaluable (NE) at that time point.

If only a subset of lesion measurements are made at an assessment, usually the case is also considered NE at that time point, unless a convincing argument can be made that the contribution of the individual missing lesion(s) would not change the assigned time point response. This would most likely happen in the case of PD.

11.4. Appendix 4 National Cancer Institute Common Terminology Criteria for Adverse Events (NCI CTCAE) Version 5.0

The NCI CTCAE Version 5.0 can be found by going to the following web site: http://ctep.cancer.gov/reporting/ctc.html

Inquiries specifically regarding the Common Toxicity Criteria (CTC) should be addressed to: ncictephelp@ctep.nci.nih.gov
