# Supplementary material for: Gallium-68-labeled fibroblast activation protein inhibitor-46 PET in patients with resectable or borderline resectable pancreatic ductal adenocarcinoma: A phase 2, multicenter, single arm, open label non-randomized study protocol
Source: PLoS One. 2023 Nov 27;18(11):e0294564. doi: 10.1371/journal.pone.0294564 (PMC10681241; doi:10.1371/journal.pone.0294564)
Supplement: S2 File — (PDF) [file pone.0294564.s003.pdf]

SOFIE
From start to clinic

July 12, 2023

To whom it may concern:

This is to confirm that the study “Gallium-68-labeled fibroblast activation protein inhibitor-46 PET in patients with resectable or borderline resectable pancreatic ductal adenocarcinoma: A phase 2, multicenter, single arm, open label non-randomized study” NCT05262855, is funded in full by Sofie Biosciences, Inc.

Thank you

Sherly Mosessian

Sherly Mosessian, Ph.D.

Chief Scientific Officer
