# Supplementary material for: Gallium-68-labeled fibroblast activation protein inhibitor-46 PET in patients with resectable or borderline resectable pancreatic ductal adenocarcinoma: A phase 2, multicenter, single arm, open label non-randomized study protocol
Source: PLoS One. 2023 Nov 27;18(11):e0294564. doi: 10.1371/journal.pone.0294564 (PMC10681241; doi:10.1371/journal.pone.0294564)
Supplement: S3 File — (PDF) [file pone.0294564.s004.pdf]

MAYO CLINIC

Principal Investigator Notification:

From: Mayo Clinic IRB
To: Ajit Goenka
CC: Britten Block
Jessica Brunn
Kurt Kevin Degillo
Kera Delaney
Kelly Dunagan
Ajit Goenka
Lori Lutzke
Casey McAdam
Maggie Olson
Kristina Schmidtknecht
Amber Stephan
Patricia Woodrum

Re: IRB Application # 22-003295

Application Title: A Phase 2, Multicenter, Single Arm, Open Label Non-Randomized Study of [68Ga]FAPI-46 PET in Patients With Resectable or Borderline Resectable Pancreatic Ductal Carcinoma

Please note that all correspondence (modifications, continuing reviews, reportable events) related to this application must be submitted electronically in the IRBe system.

The following is an excerpt from the minutes of the Mayo Clinic Institutional Review Boards (IRB-C) meeting dated 8/5/2022:

DECISION: The Committee reviewed and approved the above referenced application and noted that all requirements for approval of research (45CFR46.111 and 21CFR56.111) were met. The Committee determined that the research involves greater than minimal risk. This approval is valid for one year unless during that time the IRB determines that it is appropriate to halt or suspend the study earlier. IRB approval will expire on August 4, 2023. The Committee approved the accrual of 20 adult subjects from a screening population of 60. The Committee approved the following site to conduct the study activities as specified in the application: Mayo Clinic in Rochester, Minnesota.

REVIEW: The Committee noted receipt of the protocol GaFAPI-2022P2, Version 3.0, dated March 8, 2022. The Committee agreed that the Investigator's Data Safety Monitoring Plan was appropriate for the study. Funding for the study is provided by SOFIE. The Committee noted the IND #151901 for the study drug, 68Ga-FAPI-46, by holder, Sofie, Inc. dated November 5, 2021. The Committee reviewed the Investigator's Brochure, Version 1.0, dated October 1, 2021, for the study drug, 68Ga-FAPI-46. The Committee noted receipt of the Third Party Risk Management Approval dated May 4, 2022; Imaging Manual, Version 2, dated March 8, 2022; Laboratory Manual, Version 2, dated March 8, 2022; and Technical Operations Manual: PET, Version 1.0, dated

April 1, 2022. The Committee approved remuneration up to $30 per visit for screening and imaging visits. The Committee noted reimbursement as specified in the consent form and application will be provided.

CONTACT MATERIALS: The Committee approved the phone script and email script as submitted.

CONSENT: The Committee approved the consent form (00) as written. The final approved consent form will be provided under the Documents tab of the main study workspace in IRBe. The Committee noted that the consent process includes the use of on-site and remote electronic consent.

REMINDERS: The Committee:

- Advises the Investigator to contact Legal Contract Administration regarding an appropriate agreement(s).
- Reminds the investigator that biological specimens collected for this research study cannot be stored for future unspecified research by a commercial entity in accordance with Mayo Clinic policy.
- Reminds the investigator to submit a continuing review report prior to the expiration date (reminder will be sent prior to expiration).

Attachments (if applicable):

name	version	dateCreated	dateModified
There are no items to display			

Meissner, Irene M.D., Chair
Nicole Ritacca , Correspondent
Mayo Clinic Institutional Review Boards

IRB-C

Setup

Setup

Great, let's create your submission for a new study.

The details below will help us identify your submission. We'll continuously save your progress as you're working, and you may access this submission under "Drafts" on your dashboard.

Protocol Information

Submission Name *

GaFAPI-2022P2

Info

The submission name should be a short summary of the submission that is easy for you to reference

Protocol Title *

A Phase 2, Multicenter, Single Arm, Open Label Non-Randomized
Study of [68Ga]FAPI-46 PET in Patients with Resectable or Borderline Resectable Pancreatic Ductal Carcinoma

Sponsor *

SOFIE

Sponsor Protocol Number *

GaFAPI-2022P2

Setup

Setup

Are you also submitting a PI or site with this new study?

Please select yes or no. *

- (●) Yes
- ( ) No

Setup

Setup

Tell us a bit more about your submission, and we'll tell you what you need for board review.

Providing this information now will allow us to tell you what forms and documents are required in your submission as accurately as possible.

Recruitment Bonuses

Recruitment bonuses are extra payments tied to the rate or timing of recruitment or enrollment.

Will the Principal Investigator (PI) or research team be offered recruitment bonuses? *

- ○ Yes
- ● No

Financial Interest Disclosure

Does the Principal Investigator (PI), the PI's immediate family, or any other research personnel or their immediate families, have any of the following financial interests in any entity that is sponsoring the research, or an entity that is manufacturing the product or service being tested, not reported to this IRB in previous submissions for this protocol?

- Any **remuneration** from the entity in the previous twelve months that exceeds $5,000, when aggregated for the individual and their immediate family
- Any **equity interest** in the entity
- Any **intellectual property rights** and **interests**
- Any **governance** or **executive relationship** with the entity

* ○ Yes
* ● No

Principal Investigator

Principal Investigator

Add Principal Investigator information

First Name

Brandon

Middle Name

Last Name

Mancini

Suffix

Degrees

MD, MBA, FACRO

Email

brandon.mancini@bamfhealth.com

Phone

734-788-2254

Address Information

Company/Institution/Organization

BAMF Health I, PC

Country

United States

Address Line 1

109 Michigan St, NW

Address Line 2

Suite 200

City

Grand Rapids

State

Michigan

Postal Code

49503

Principal Investigator (PI) Specialty

Is the Principal Investigator (PI) a physician?

- (selected) Yes
- No

National Provider Identifier (NPI) #

1366731838

Look up NPI# at https://npiregistry.cms.hhs.gov

What are the specialties of the site?

Primary specialty

Radiation Oncology

Principal Investigator (PI) Licensure

Does the Principal Investigator have a medical license?

- (•) Yes
- ( ) No

Are all medical licenses on file with the IRB?

- ( ) Yes
- (•) No

Warning

Because the Principal Investigator (PI) has a medical license:

Submit copies of all current medical licenses showing the issuing authority, license number, and expiration date.

Contacts

Contacts

Are there any designated contacts for this research?

- (•) Yes
- ( ) No

Add contacts here for users who will be:

- main contacts for questions from WCG IRB staff
- main contacts for external review notifications
- listed on the Certificate of Action

Contacts

Contact Type

Study Coordinator

Prefix

First Name	Mark

Last Name	Olson

Suffix

Email	mark.olson@bamfhealth.com

Phone	847-814-5338

Company/Institution/Organization

BAMF Health

Contact Type

Study Coordinator

Prefix

First Name	Dan

Last Name	Rogers

Suffix

Email	dan.rogers@bamfhealth.com

Phone	847-814-5338

Company/Institution/Organization

BAMF Health

Contact Type

Sponsor

Prefix

First Name	Bridget

Last Name	Adams

Suffix

Email	bridget.adams@sofie.com

Phone	3194301192

Company/Institution/Organization

SOFIE

Initial Review Questionnaire

Initial Review Questionnaire

For clinical use of a Humanitarian Use Device (HUD), Expanded Access, Compassionate Use, and Emergency Use, see separate application forms on the IRB Web site.

Multi-site Studies Central IRB

For multi-site studies - has the sponsor/CRO designated this IRB as the central IRB for most sites or the single IRB for this study?

- ○ Yes
- ● No

Contract Research Organization (CRO) Information

Is a Contract Research Organization (CRO) involved in the research?

- ● Yes
- ○ No

Contract Research Organization (CRO) name

MMS Holdings

Subject payment for investigational product?

Will subjects be required to pay for the investigational product? If yes, how much?

- ○ Yes
- ◉ No

Federal Funding

Is this research funded, supported, or conducted by a United States federal department or agency?

- ○ Yes
- ◉ No

Phase One - Healthy Subject Clinical Trials

Is this submission for a Phase One - Healthy Subject Clinical Trial?

- ○ Yes
- ◉ No

Clinical Trial Information

Will you or others post the research on ClinicalTrials.gov?

- ◉ Yes
- ○ No

ClinicalTrials.gov Identifier

NCT05262855

Will you or others submit data from this research to the US Food and Drug Administration (FDA) or hold data from this research for inspection by the FDA?

- ◉ Yes
- ○ No

This research is for a clinical trial of a... (select all that apply):

- [ ] Device
- [x] Drug
- [ ] Other

Are you conducting the research under an Investigational New Drug Application (IND)?

- ◉ Yes
- ○ No

If the IRB has an Investigator Brochure on file that is more current than the one posted on-line or submitted with this application, the IRB will review the research using the more current Investigator Brochure on file.

IND #

151901

Drug Name(s)

68GaFAPI-46

The IRB should use the Investigator Brochure(s) that:

- ( ) Is on-line
- (x) Is submitted with this application
- ( ) The IRB has on file

Warning

Because you have indicated that the Investigator's Brochure is being submitted with this application:

Submit the Investigator's Brochure(s) for the investigational drug(s).

Does the investigator hold the IND?

- ( ) Yes
- (x) No

Vulnerable Populations

Select all populations your research will involve, if any. Leave all boxes unchecked if none apply.

- [ ] Subjects who are adults unable to consent
- [ ] Subjects who are children
- [ ] Children who are wards of the state
- [ ] Non-viable neonates
- [ ] Neonates of uncertain viability
- [ ] Subjects who are prisoners
- [ ] Subjects who are pregnant at enrollment
- [ ] Subjects who become pregnant while on study

Other Populations

Will the research involve subjects who are students or employees of the investigators?

( ) Yes (x) No

Prior IRB Review

Has another IRB reviewed this research or site and decided to table, defer, disapprove, suspend, terminate, or decline to approve it?

( ) Yes (x) No

IRB Transfer

Are you transferring IRB oversight from another IRB to this IRB?

( ) Yes (x) No

Environmental Protection Agency (EPA) Oversight

Will you or others submit data from this research to the US Environmental Protection Agency (EPA)?

○ Yes ● No

Human Gene Transfer

Does this research involve any form of human gene transfer as described in Section III-C of the NIH Guidelines?

○ Yes ● No

Committee Requirements

Does this study require an independent Data Monitoring Committee / Data Safety Monitoring Board?

○ Yes
● No

Does this study require an independent Endpoint Adjudication Committee?

○ Yes
● No

Consent

Will subjects or their representatives provide informed consent to take part in this research?

● Yes
○ No

Consent Setting

Indicate the setting of the consent process:

Private room

Consent Process

Will the research team do all the following?

- Give the person providing informed consent as much time as they need to decide.
- If the person providing informed consent needs more time than is allowed by the research design, not enroll the prospective subject.
- Evaluate whether the person providing informed consent is experiencing time pressure to decide, and if so, do not enroll the prospective subject, even if the person providing informed consent agrees to be in the research.
- Ensure there is no threat of harm or adverse consequences to the prospective subject for a decision to not take part in the research.
- Stop the informed consent process once the person providing consent indicates that he or she does not want to take part in the research.
- Evaluate whether the person providing informed consent is being coerced or unduly influenced by others to take part in the research, and if so, not enroll the prospective subject, even if the person providing informed consent agrees to be in the research.
- Communicate in the preferred language of the person providing informed consent.

• Adapt the presentation of the information to the subject's capacities in terms of intelligence, rationality, maturity and language.

• Invite and answer questions from the person providing informed consent.

• Evaluate whether the person providing informed consent understands the information provided, and not enroll a prospective subject who does not understand, even if that person providing informed consent agrees to be in the research.

• Ensure that no information is provided to the prospective subject or the person providing informed consent that is made to waive or appear to waive any of the prospective subject's legal rights, or releases or appears to release the investigator, the sponsor, the institution or its agents from liability for negligence.

• Communicate to the person providing informed consent all the information in the consent document or script approved by the IRB.

• Not enroll a prospective subject when the person obtaining informed consent is unwilling to listen to or consider the information, even if the person providing informed consent agrees to be in the research.

- (●) Yes
- ( ) No

Consent Documentation

Would you like WCG to maintain a Protocol-Level Template Consent Form?

- (●) Yes
- ( ) No

Will subjects or their representatives sign a written consent form?

- (●) Yes
- ( ) No

How will signatures be obtained?

- (●) Wet Ink
- ( ) Electronic
- ( ) Both

Consent Form Processing

Does your organization have pre-approved consent language on file with the IRB?

- ( ) Yes
- (●) No

Indicate how you want us to process consent forms:

- ( ) The IRB should insert the pre-approved consent language on file for my Institution and the site-specific contact language provided in this submission form into the most recent IRB-approved consent template. (If you include a consent form with this submission, the IRB will not use it if there is a template on file.)
- (●) The IRB should add site-specific contact language provided in this submission form to the currently approved template. (If you include a consent form with this submission, the IRB will not use it if there is a template on file.)
- ( ) I am submitting a consent with requested language changes shown as tracked changes
- ( ) Other

Consent Documentation Process

Will the research team do all the following?

- The investigator will give the person providing consent adequate opportunity to read the consent document before it is signed and dated
- The consent document will be signed and dated by the person providing consent
- The consent document will be signed and dated by the person obtaining consent
- A signed and dated copy of the consent document will be given to the person providing consent
- For a clinical trial: If the person providing consent cannot read, an individual who is independent of the trial, who cannot be unfairly influenced by people involved with the trial ("impartial witness") will be present during the entire informed consent discussion and will sign and date the consent document to attest that the information in the consent document and any other written information was accurately explained to, and apparently understood by, the person providing consent.

- (●) Yes
- ( ) No

HIPAA Waiver of Authorization

What type of waiver of HIPAA authorization, if any, are you requesting?

- ( ) Full waiver of authorization
- ( ) Partial waiver of authorization for access to records for subject recruitment or screening
- ( ) Partial waiver of authorization for waiver of signing an authorization form
- (●) None

Subject Payment

Will you pay Subjects for participation?

- (●) Yes
- ( ) No

Provide Subject payment language using one of the methods described below:

- (●) I have already incorporated subject payment language into documents submitted with this application
- ( ) I will provide subject payment information in this form for the IRB to incorporate into submitted documents

List the submitted documents that include payment language:

Informed Consent Form (ICF)

Secondary Research

Select One:

- ( ) There is a possibility that identifiers might be removed from the identifiable private information or identifiable biospecimens, and after such removal, the information or biospecimens may be used for future research studies or distributed to another investigator for future research studies without additional informed consent from the subject or the legally authorized representative.
- (●) Subject information or biospecimens collected as part of the research, even if identifiers are removed, will not be used or distributed for future research studies.
- ( ) The research does NOT involve the collection of identifiable private information or identifiable biospecimens.

Confidentiality

Confidentiality refers to the agreements regarding how data will be managed and used.

Will you be subject to and in compliance with HIPAA?

- (selected) Yes
- No

Will the research be covered by a Certificate of Confidentiality (COC)?

- Yes
- (selected) No

Additional Methods to Maintain Confidentiality

Describe any additional procedures to protect confidentiality: (e.g., confidentiality agreements, coding)

All subjects will be assigned a study number and only the site will have information linking the PHI to the study number. All information submitted to the sponsor or third part vendors will be de-identified.

Institutional Services

Will you conduct this research through an organization that has a contract or Master Services Agreement (MSA) to use WCG IRB for IRB Services?

- (selected) Yes
- No

Name of organization relying on WCG IRB (if known)

BAMF Health, Inc.

WCG IRB Institution # of organization relying on WCG IRB (if known)

Site Management Organization (SMO) Information

Is a Site Management Organization (SMO) involved with this research site?

- Yes
- (selected) No

Site Enrollment Estimate

The IRB will not consider this estimate to be an enrollment limit for the site.

What is the planned number of subjects to be enrolled locally?

20

Research Team Information

Indicate the number of investigators and research staff involved with the conduct this research:

Physician Sub/Co-investigators

1

Other Sub/Co-investigators

1

Research Coordinators

2

Other research staff

Principal Investigator (PI) Experience

How many clinical trials is the PI currently conducting?

2

Among the clinical trials that the PI is currently conducting, how many are open to enrollment?

2

Research Team Training

The Principal Investigator (PI) must ensure that all investigators and research staff undergo training on the ethics and regulations of human subject protections before being involved in the conduct of this research. For clinical research, the Principal Investigator (PI) must ensure that all investigators and research staff undergo training on Good Clinical Practice (GCP).

- Have all investigators and research staff involved with the conduct of this research taken one or more of the following programs and all applicable training programs noted as required?
    - ACRP Certified Clinical Investigator Training
    - CenterWatch: Protecting Study Volunteers in Research
    - Collaborative IRB Training Initiative (CITI)
    - DIA Certified Investigator (CCI)
    - SOCRA Clinical Research Professional (CRP)
    - Tri-Council Policy Statement online training (TCPS)
    - WCG Academy
    - WCG InvestigatorSpace® Training

(●) Yes
( ) No

Subject Privacy

Privacy refers to persons' interest in controlling the access of others to themselves, such as the ability to control who sees them, hears them, touches them, and has access to their private information. Additional privacy interests include the time and place where individuals provide information, the nature of the information provided by the individuals, the nature of the individual's experiences during the trial, and who receives and can use the information.

Will you or others perform procedures in a private setting?

- (selected) Yes
- ( ) No

Administrative Actions

Has the Principal Investigator (PI) or any other personnel involved in this research had any of the following that has not been reported to this IRB:

- FDA Warning Letter
- NIDPOE (Noticed of Initiation of Disqualification Proceedings and Opportunity to Explain)
- Suspension or termination by an IRB
- Suspension by a federal or governmental agency (such as FDA, HHS, or Health Canada)
- OHRP Determination Letter, Health Canada Inspection Letter with observations, or similar
- Form FDA 483 in the past 5 years

- OR -

Has the Principal Investigator (PI) or any other personnel involved in this research had any of the following denied, revoked, suspended, reduced, limited, placed on probation, not renewed, relinquished, sanctioned, fined, or subject to disciplinary action that has not been reported to this IRB?

- Clinical privileges at any site
- DEA licensure
- Fellowship/board certification
- Medical licensure in any state, nation, or province
- Membership on any hospital staff
- Prescribing privileges
- Professional sanctions including fines and public reprimands
- Professional society membership
- Research privileges at any site

- OR -

Is there any action or investigation currently pending before any court of law, federal agency, or state licensing board concerning the professional conduct of the Principal Investigator (PI), or any other personnel involved in this research in that individual's capacity as a research investigator or as a clinician that has not been reported to this IRB?

- ( ) Yes
- (selected) No

Required Submission Materials for Protocol and Site Submission

To avoid processing delays, remove security/password protection from all submitted documents.

Submit the following documentation:

- Final protocol (or most recent version with any applicable amendments)
- Supporting documents
- All information intended to be seen or heard by subjects, including:
- Consent documents (in Microsoft Word compatible format)
- Information sheets (in Microsoft Word compatible format)
- Advertisements and recruitment scripts (Advertisements and recruitment materials and change to advertisements and recruitment materials must be IRB approved before their use)
- Curriculum vitae for the Principal Investigator (PI), if a current one is not already on file with the IRB

Research Location

Physical address where subjects will be seen or research will take place:

Locations

Company/Institution/Organization

BAMF Health

Country

United States

Address Line 1

151901

Address Line 2

Suite 100

City

Grand Rapids

State

Michigan

Postal Code

49503

Which of the following best describes this location's function?

Medical Office or Research Clinic

Describe any additional resources available at this location that are relevant to this research: (optional)

Site number assigned by sponsor (optional)

005

Daytime phone number for subjects to call for questions or injury

616-330-3886

24 hour phone number for subjects to call for questions or injury

888-870-8998

Do any communities around the above locations(s) have a negative attitude towards the conduct of research?

- ○ Yes
- ● No

Does a local IRB have jurisdiction over research over any of the above locations? (If this site is covered by a Master Services Agreement (MSA) or is a member of our Global Research Network (GRN), you may check "No")

- ○ Yes
- ● No

Are there any state or local laws that impose additional requirements for research?

- ○ Yes

- [x] No

Is the distance between any location and the main location greater than 50 miles (80 kilometers)?

- [ ] Yes
- [ ] No
- [x] There is only one location

Billing Information

How should we send invoices to the billing contact?

- [x] Email
- [ ] Printed mail

Mail stop/cost center (if applicable)

Purchase order number (PO#) (if applicable)

sIRB code (if applicable)

Prefix

First Name

Bridget

Last Name

Adams

Suffix

Email

bridget.adams@sofie.com

Phone

319-430-1192

Company/Institution/Organization

SOFIE

Country

United States

Address Line 1

21000 Atlantic Boulevard

Address Line 2

Suite 730

City

Dulles

State

Virginia

Postal Code

20166

Translations

If you need translated documents, please wait to submit the documents until the Certificate of Action is received. WCG IRB will not process any translation request until after the English versions are received.

Special Instructions

Provide any special instructions or additional relevant information for this submission:

NYU Langone Health Research Navigator MyStudies

Date: Monday, August 28, 2023 10:05:35 AM Print Close

View: SF - Subject & Analysis Information

Conditions Being Studied

1.0	* **Primary Condition/Disease (Text Entry):** Pancreatic Ductal Adenocarcinoma
1.1	**Primary Condition/Disease (Coded Entry):**
2.0	**Secondary Conditions/Diseases (Text Entry):**
2.1	**Secondary Conditions/Diseases (Coded Entry):** ID Name Type — There are no items to display

Eligibility

1.0	* **Gender:** Both (or n/a)
2.0	* **Minimum Age:** 18 years
3.0	* **Maximum Age:** 100 years
4.0	* **Study includes Healthy Volunteers:** ○ Yes ◉ **No**
5.0	For each vulnerable population that is indicated, be sure to fill out and complete the IRB's appropriate Vulnerable Populations appendix, which can be found here: Link<br>* **Vulnerable populations included:**<br>No Vulnerable Populations
5.1	Please make sure your study involves employees as referenced in the NYU Langone HRPP and IRB Policy and Procedure Manual found at Instituional Review Board Policies, Structure & Accreditation | NYU Langone Health<br><br>Do not check employee or student if you think a subject who enrolls for your study may also be an employee or student. **You would ONLY CHECK** Employees and Students **WHEN** the research is designed to focus on or study Employees and/or Students and/or where the research contemplates recruitment using Direct Recruitment methods to recruit and enroll Employees and/or Students, additional measures are required to ensure that their participation in the research is entirely voluntary and that their decision-making is freely-informed.

STUDENTS
graduate students, medical students, residents or fellows, post-doctoral fellows: ◯ Yes ◉ **No**
PhD candidates: ◯ Yes ◉ **No**

EMPLOYEES (not including NYU-paid students above)
NYU Grossman School of Medicine or NYU Langone Health employee:
◯ Yes ◉ **No**

5.2 **Employee Details**

Above you indicated NYU employees will be involved in your research. Please answer the following questions with appropriate detail to allow for a full review by the Human Resource Department. Your answers will be used to assess whether or not employees may be included in your study. Failure to completely answer the questions below will delay your study's review.

Describe the category of employees you plan to recruit as subjects, for example if the employee category is limited (e.g., radiologists, nurses, etc.), explain that here:

Describe whether the employee population is to be limited to a unit, clinical service, campus, etc.,:

Describe the types of recruitment to be used in this study for recruitment of employees (PLEASE NOTE: Broadcast email as a recruitment method is not permitted under NYU Langone Human Research Protection and IRB policy). Include "listserv", "posters/flyers" and any other recruitment media you are planning to use:

If this research includes procedures, questions, surveys or any processes that requests or collects information from employees that could be considered sensitive (ie: Family Planning, Sexual Orientation, etc.), describe that here (or state that this study does not include sensitive information):

6.0 * **Key Inclusion Criteria:**
Pathologically confirmed pancreatic ductal adenocarcinoma
Treatment-naïve
Staged as resectable or borderline-resectable
Planned to undergo surgical resection or to receive neoadjuvant therapy (i.e., chemotherapy, radiation therapy, or combination) and subsequent possible surgical resection
Anatomic imaging (e.g., CT, MRI) obtained within ≤ 28 days of consent
Age ≥ 18 years
Completed informed consent as determined per the IRB of record

7.0 * **Key Exclusion Criteria:**
Pregnant as determined by a pregnancy test as per institutional guidelines for individuals of child-bearing potential
Declining to use effective contraceptive methods during the study (for individuals of child-producing potential)
Need for emergent surgery that would be delayed by participation
Bacterial, viral, or fungal infections requiring systemic therapy
Serious co-morbidities and serious nonmalignant disease (e.g., hydronephrosis, kidney failure, liver failure, systemic or local inflammatory or autoimmune diseases or other conditions) that in the opinion of the investigator, physician of record and/or Sofie could

	compromise patient safety and/or protocol objectives.<br>Known diagnosis of autoimmune disorders<br>Patients receiving any other investigational agent within the past 28 days<br>Breastfeeding. Note: nursing parents are allowed if the potential participant commits to pumping breast milk and discarding it from injection to ≥ 24 hours from the time of the [68Ga]FAPI-46 injection.<br>Known hypersensitivity to any excipients used in [68Ga]FAPI-46: trace amounts of sodium acetate sodium ascorbate and/or hydrochloric acid

Enrollment

**1.0**	* **Projected number of subjects to sign consent - NYU PI:**<br>40
**2.0**	* **Projected Accrual - NYU PI:**<br>10
**3.0**	* **Projected Accrual - All sites:**<br>60
**4.0**	**Does the protocol include an in-patient component?** ○Yes ◉ **No**
**5.0**	* **Enrollment Duration:** 24 Month(s)<br>This is the amount of time you anticipate needing to reach your projected accrual goal at NYU. This field is also reported to the Dean’s Dashboard. See here for additional information.
**6.0**	**Anticipated number of screen failures:**
**7.0**	**Anticipated Study Enrollment End Date:**

Primary & Secondary Outcome Measures

**1.0**	* **Primary Outcome:**<br>Performance [sensitivity, specificity, accuracy] of [68Ga]FAPI-46 PET imaging to detect FAP-expressing cells, using histopathology as truth standard.
**2.0**	* **Secondary Outcomes:**<br>Positive and negative predictive values, as well as accuracy of [68Ga]FAPI-46 PET images, to detect FAP-expressing cells using histopathology as truth standard.<br>Histopathology with FAP staining on FAP IHC assay.<br>Number of participants with treatment-related adverse events as assessed by CTCAE v5.0<br>[68Ga]FAPI-46 accumulation observed in local and metastatic disease compared to radiological (i.e. CT, MR) and/or 18F-FDG PET.<br>[68Ga]FAPI-46 accumulation observed by (PET)/ (CT) pre and post in patients undergoing Neoadjuvant treatment.

iConnect Information

**1.0**	**Do you want this study included in the iConnect clinical studies registry on the NYU Langone Health website?**<br>◉ **Yes** ○No

Date: Monday, August 28, 2023 11:17:17 AM

Print Close

ID: IRB#22-000631 View: NEW 9.6 - Use of Data and/or Specimens without Direct Contact

Warning: Save your work at least every 15 minutes by clicking "Save" or "Continue."

Use of Data and/or Specimens without Direct Contact

You indicated that some or all of the research activities do not involve direct contact with study participants (Section 8.1/item 1.0). Please provide the following information.

1.0 If **all** of your research activities are without direct contact with study participants, provide the following information:

1.1 **Indicate the purpose of the research, specifying the problems and/or hypotheses to be addressed**:

1.2 **Describe the study design and proposed data analyses**:

1.3 ***If you will conduct genetic analysis with specimens, provide your assurance that the results will not be disclosed to subjects or used for clinical care.**

- ○ Agree
- ◉ **Not Applicable**

2.0 ***Describe specimens and/or data that will be acquired without direct contact with study participants. Complete this item for each type used in the study:**

	**Source**	**Data and/or Specimens Information**
View	UCLA	

**Data and/or Specimens? Indicate all that apply:**	Specimens
**Indicate whether the data and/or specimens are pre-existing, at the time of this study, and/or if collection will be prospective:**	Prospective
**Describe the data and/or specimens and indicate the original collection dates:**	Specimens will be paraffin embedded pancreatic cancer tissue that we will perform IHC for FAP expression Planned dates: 8/2022 - 2/2023
**Indicate the approximate number of data records and/or specimens to be collected:**	20
**Will the specimens be used with animals?**	No
**If yes, indicate the IACUC Number:**	*No Value Entered*

3.0 ***If any sources of data and/or specimens are not at UCLA, provide your agreement that the appropriate institutional approvals for release will be obtained** (e.g., IRB approval).

- ○ Agree
- ◉ **Not Applicable**

If you plan to send UCLA Health data to third parties, please contact the CTSI for directions about additional requirements. https://www.ctsi.ucla.edu/researcher-resources/pages/third_party

4.0 **Attach any data abstraction tools or lists with the data elements to be collected.**

Document Name	Document Version #
There are no items to display	

If you will access information from UCLA Health records, attach a copy of your completed UCLA EHR Data Abstraction template for research. For more information see: https://ctsi.ucla.edu/researcher-resources/pages/datarequests

ID: IRB#22-000631 View: Specimens and/or data that will be acquired without direct contact with study participants

Specimens and/or Data that will be Acquired without direct contact with study participants

1.1 ***Data and/or Specimens? Indicate all that apply:**

- [ ] Data
- [x] **Specimens**

1.2 ***Indicate the source of the data and/or specimens. If the source is UCLA or a previous study, also indicate the IRB#:**

UCLA

1.3 ***Indicate whether the data and/or specimens are pre-existing, at the time of this study, and/or if collection will be prospective. Check all that apply:**

- [ ] Pre-existing
- [x] **Prospective**

1.4 ***Describe the data and/or specimens and indicate the original collection dates. If collection is in progress, indicate the planned end date or "continuing."** (*e.g., academic records for children 6-12 years for the time period between 1995-2005, or tumor samples collected from adults between January 1, 2009 to December 31, 2009*).

Specimens will be paraffin embedded pancreatic cancer tissue that we will perform IHC for FAP expression

Planned dates: 8/2022 - 2/2023

1.5 ***Indicate the approximate number of data records and/or specimens to be collected.**

20

1.6 **If you indicated that you will be using specimens, provide the following information.**

1.6.1 **Will the specimens be used with animals?**

○ Yes ◉ **No**

1.6.1.1 **If yes, indicate the IACUC Number:**
