# Supplementary material for: Gallium-68-labeled fibroblast activation protein inhibitor-46 PET in patients with resectable or borderline resectable pancreatic ductal adenocarcinoma: A phase 2, multicenter, single arm, open label non-randomized study protocol
Source: PLoS One. 2023 Nov 27;18(11):e0294564. doi: 10.1371/journal.pone.0294564 (PMC10681241; doi:10.1371/journal.pone.0294564)
Supplement: S4 File — (DOCX) [file pone.0294564.s005.docx]

**Appendix 1**

**FAPα Immunohistochemistry (IHC) Protocol and Proof-of-Concept Results**

Archival formalin fixed paraffin embedded (FFPE) pancreatic tumor cases were randomly chosen from the Discovery Life Sciences tissue bank (Discovery Life Sciences, Newtown, PA, USA). These tumor tissues were ethically acquired from various clinical sites with diverse patient populations with personal identifiers redacted.

The FFPE tumor tissues were sectioned (4–5 μm) onto positivity charged slides (Superfrost Plus Microscope Slides; [Cat No. 12-550-15] Fisher Scientific, Pittsburgh, PA, USA) and dry heated for 1 h at 65°C within 2 days of testing. Slides were loaded in racks and placed into the PT link (Dako/Agilent, Santa Clara, CA, USA) in pre-heated (65°C) 1x High pH Target Retrieval Solution (included in the EnVision FLEX+, High pH (Link) Kit, [Cat No. K800021-5], Dako/Agilentto undergo deparaffinization, rehydration and epitope retrieval for 20 minutes at 97⁰C. After cooling to 65⁰C, racks were rinsed with 1x FLEX wash buffer.

All procedures were automated at room temperature using the Dako Autostainer Link 48 platform. EnVision FLEX High pH (Link) Kit from Dako/Agilent was used for IHC detection and stored ready-to-use at 2-8°C. The EnVision FLEX+ Kit included FLEX Wash Buffer, FLEXPeroxidase-Blocking Reagent, FLEX HRP (horseradish peroxidase), and FLEX DAB+ Substrate Buffer/Chromogen. Proteinase K ([Cat No. S302080-2], Dako/Agilent) was diluted 1:40 in Tris Buffered Saline with 0.02% (v/v) Tween-20 detergent (TBST) (Fisher Scientific). Rabbit monoclonal antibody anti-FAPα (SP325) [Cat No. ab227703], Abcam, Boston, MA, USA) was diluted with Dako/Agilent Antibody Diluent ([Cat No. S080983-2], Dako/Agilent. DAB was prepared according to manufacturer’s instructions.

The protocol was run as follows with intervening rinses in FLEX wash buffer: 10 min Proteinase K diluted 1:40, 5 min FLEXPeroxidase-Blocking Reagent, 60 min rabbit monoclonal antibody anti-FAPα (SP325) diluted 1:200, 15 min FLEX Rabbit Linker ([Cat No. K8009], Dako/Agilent), 20 min FLEX HRP, 5 min FLEX Wash Buffer, 10 min FLEX DAB, and 2 min FLEX Hematoxylin ([Cat No. K800821-2], Dako/Agilent).

Slides were immersed in room temperature deionized water and transferred to the coverslip area. Slides were rinsed in distilled water and dehydrated with washes in an alcohol series (95%, 100% ethanol) and organic solvent (xylene, 100%, four changes). After dehydration, slides were cover slipped using non-aqueous semi-permanent mounting media ([Cat No. 22-050-262] Fisher Scientific).

In support of this study, a FAPα expression levels, detected by the validated IHC assay, were evaluated in archival, treatment-naïve, FFPE tumor samples. Tissues included 30 unique pancreatic tumor cases. Cases with at least 100 viable invasive tumor cells were scored for FAPα stromal staining within the total evaluable area of the tissue. Necrotic regions, *in situ* carcinoma, and poorly fixed regions of the tissue were excluded from scoring. Slides were scanned at 20x magnification using Aperio AT2 (Leica Biosystems, Deer Park, IL, USA). The slides were scored by a board-certified pathologist, using the image management system eSlide Manager (Version 12.4.5.5008, Aperio, Leica).

Regions of tumor nests and surrounding tumor-induced stromal were evaluated to provide percent (%) of tumor and percent (%) of stroma by area. Semiquantitative analysis of the abundance of stromal staining was assessed as 0, 1, 2, and 3 as previously established. A score of 0 was defined as the complete absence or weak FAP immunostaining in <1% of the tumor stroma; a score of 1 was given for positivity in 1% to 10% of stromal cells; a score of 2 was given for positive FAP immunostaining in 11% to 50% of stromal cells; and a score of 3 was given for positive FAP staining in >50% of stromal cells. FAPα reactivity was also scored using differential intensity scores (0, null; 1+, low or weak; 2+, moderate; 3+, high or strong). A total percentage score (% of stromal cells staining at ≥1+ intensity) was used to semi-quantitatively evaluate stromal expression of FAPα. The H-score was calculated using the following formula: H-score = [(% at 0)×0]+[(% at 1+)×1]+[(% at 2+)×2]+ [(% at 3+)×3] to generate a value ranging from 0 to 300.
